# Unraveling Radical and Oxygenate Routes in the Oxidative Dehydrogenation of Propane over Boron Nitride

**DOI:** 10.1021/jacs.2c12970

**Published:** 2023-03-03

**Authors:** Zihao Zhang, Jinshu Tian, Xiangkun Wu, Ivan Surin, Javier Pérez-Ramírez, Patrick Hemberger, Andras Bodi

**Affiliations:** † Paul Scherrer Institute, 5232 Villigen, Switzerland; ‡ College of Chemical Engineering, Zhejiang University of Technology, Hangzhou 310014, China; § Institute for Chemical and Bioengineering, Department of Chemistry and Applied Biosciences, ETH Zurich, Vladimir-Prelog-Weg 1, 8093 Zurich, Switzerland

## Abstract

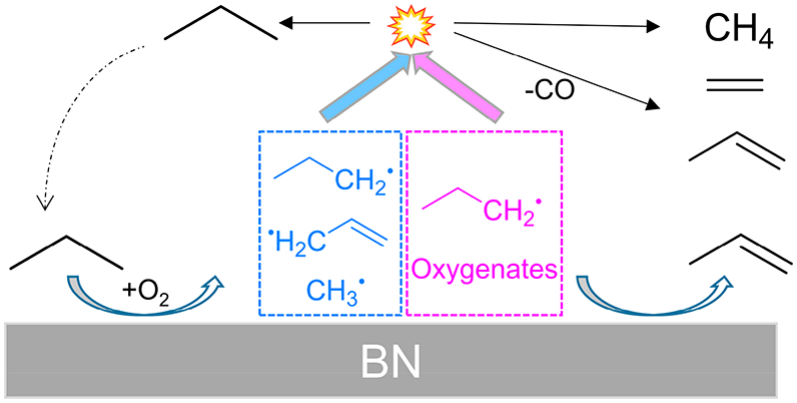

Oxidative dehydrogenation of propane (ODHP) is an emerging technology to meet the global propylene demand with boron nitride (BN) catalysts likely to play a pivotal role. It is widely accepted that gas-phase chemistry plays a fundamental role in the BN-catalyzed ODHP. However, the mechanism remains elusive because short-lived intermediates are difficult to capture. We detect short-lived free radicals (CH_3_^•^, C_3_H_5_^•^) and reactive oxygenates, C_2–4_ ketenes and C_2–3_ enols, in ODHP over BN by *operando* synchrotron photoelectron photoion coincidence spectroscopy. In addition to a surface-catalyzed channel, we identify a gas-phase H-acceptor radical- and H-donor oxygenate-driven route, leading to olefin production. In this route, partially oxidized enols propagate into the gas phase, followed by dehydrogenation (and methylation) to form ketenes and finally yield olefins by decarbonylation. Quantum chemical calculations predict the >BO dangling site to be the source of free radicals in the process. More importantly, the easy desorption of oxygenates from the catalyst surface is key to prevent deep oxidation to CO_2_.

## Introduction

Propylene (C_3_H_6_) is a crucial platform chemical in the petrochemical industry for organic and polymer synthesis. It is mainly produced by petroleum-derived steam cracking and fluid catalytic cracking.^1−3^ The annual C_3_H_6_ production was 130 Mt in 2019 and is projected to grow to 191 Mt by 2030.^4^ The recent shift from petroleum-derived naphtha to shale gas feedstock greatly increases the availability of ethylene (C_2_H_4_) and results in a gap between the supply of C_3_H_6_ and rising global demand.^5,6^ To alleviate the “propylene gap”, the nonoxidative dehydrogenation of propane (C_3_H_8_) was industrialized recently by Honeywell UOP (Oleflex) and ABB Lumus (Catofin).^7,8^ However, this process suffers from (1) rapid accumulation of coke, which requires frequent catalyst regeneration, and (2) high energy need due to the endothermicity of the process.^9,10^ Therefore, the oxidative dehydrogenation of propane (ODHP) represents a promising alternative, with an estimated energy saving of ca. 45% due to its exothermicity, as well as the prevention of coke formation in the presence of O_2_.^11−14^ Transition metal oxide catalysts, such as vanadia species (VO_*x*_), are able to activate C–H bonds in C_3_H_8_, boding well for ODHP performance.^15−18^ However, the partially occupied d-orbitals in transition metal oxides interact with the reactive intermediates, binding them strongly to the catalyst surface and leading to overoxidation to CO and CO_2_, limiting the selectivity of the process.^11,19,20^

In 2016, boron nitride (BN) emerged as a unique ODHP catalyst because of its high selectivity to C_3_H_6_ and the prevention of CO_*x*_, especially CO_2_, formation.^21^ Modified BN^22,23^ and further boron-containing catalyst candidates, e.g., boron oxides (BO_*x*_),^24−27^ were widely studied to identify the active site. The similar reaction kinetics over BN and BO_*x*_ were rationalized by surface oxygen functionalization on BN, generating BO_*x*_ sites as the effective active site in ODHP.^28,29^ The conversion was found to increase with higher dilution over boron-based catalysts, which was one of the peculiar kinetic features implying a gas-phase C_3_H_6_ formation route.^30^ Based on computational results and kinetic measurements, distinct surface-confined and gas-phase mechanisms were, thus, proposed over boron-based catalysts for ODHP.^31−33^ So far, the only experimental evidence for the existence of a gas-phase route has been the observation of the methyl radical.^34^ The gas-phase reaction mechanism and the possibility of free radical involvement beyond the methyl radical remain unknown, which makes it challenging to rationally optimize catalysts for the practical application of ODHP.

In this contribution, we shed light on the intermediates and products evolving in real time in ODHP over BN by *operando* synchrotron photoelectron photoion coincidence (PEPICO) spectroscopy. *Operando* PEPICO detects short-lived reactive intermediates, e.g., radicals, ketenes, and enols, desorbed from the catalyst surface^35,36^ and, thus, provides direct experimental evidence to understand the ODHP reaction network over BN. A silica-supported vanadia catalyst (VO_*x*_/SiO_2_) was also studied for direct comparison with the catalytic mechanism over BN. Temperature-dependent ODHP experiments in a tubular continuous-flow reactor coupled to the PEPICO endstation also provide temperature profiles of stable species and reactive intermediates. Surface-confined density functional theory (DFT) calculations on boron active sites and gas-phase G4 calculations reveal the formation mechanism of short-lived radical intermediates detected by *operando* PEPICO spectroscopy. A reaction mechanism for boron-catalyzed ODHP is proposed, encompassing coupled surface-confined and gas-phase reaction steps.

## Results and Discussion

### ODHP as a Function of Temperature over BN and VO_*x*_/SiO_2_

**Figure 1:**
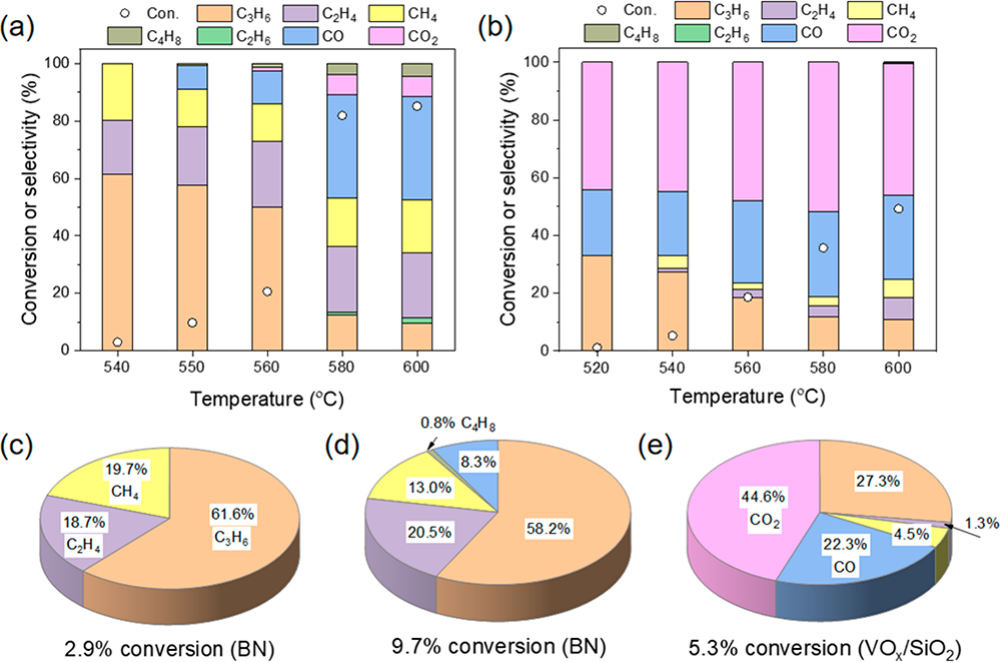
Influence of reaction temperature on catalytic ODHP performance over (a) BN and (b) VO_*x*_/SiO_2_; the product selectivity over BN at C_3_H_8_ conversions of (c) 2.9% and (d) 9.7%, as well as over VO_*x*_/SiO_2_ at C_3_H_8_ conversion of (e) 5.3%. Reaction conditions: 20 mg catalyst, feed: 10% C_3_H_8_ and 20% O_2_ balanced in Ar; 66 mL/min total flow rate.

The temperature-dependent ODHP performance over commercial BN and homemade VO_*x*_/SiO_2_ catalysts is compared in Figure 1. Over the BN catalyst, the main products are propene, ethene, and methane at temperatures below 560 °C with the propane conversion lower than 21% (Figure 1a). Small amounts of CO and butene isomers (C_4_H_8_) are also found at 550–560 °C. When increasing the temperature so that the C_3_H_8_ conversion rises to above 80%, the selectivity to C_3_H_6_ decreases with increased formation of CO, CO_2_, and C_4_H_8_. In contrast, the main products over a VO_*x*_/SiO_2_ catalyst are C_3_H_6_ and CO_*x*_ (Figure 1b), the latter being predominant at high C_3_H_8_ conversion. This indicates that overoxidation can be curtailed using the BN catalyst, especially at low C_3_H_8_ conversion. As a result, the total selectivity to hydrocarbons (C_3_H_6_, C_2_H_4_, and CH_4_) over BN is significantly higher than over VO_*x*_/SiO_2_ at a comparable C_3_H_8_ conversion. For example, the selectivities to C_3_H_6_, C_2_H_4_, and CH_4_ are 61.6%, 18.7%, and 19.7%, respectively, at a C_3_H_8_ conversion of 2.9% over a BN catalyst (Figure 1c), which corresponds to essentially full selectivity to hydrocarbons. Increasing the C_3_H_8_ conversion to 9.7%, the selectivity to C_3_H_6_, C_2_H_4_, and CH_4_ is still ca. 92%, while 0.8% C_4_H_8_ and 8.3% CO are also formed over BN (Figure 1d). This can be compared with only 27.3% selectivity to C_3_H_6_ with dominant production of CO and CO_2_ already at 5.3% conversion over VO_*x*_/SiO_2_ (Figure 1e).

The selectivity to CO_2_ is always low over BN, and CO is the main overoxidized product at high temperatures, as opposed to the VO_*x*_/SiO_2_ catalyst, where CO_2_ dominates. Another difference between the BN and VO_*x*_/SiO_2_ catalysts is that small amounts of C_4_H_8_ isomers are only observed over BN. The product distributions over BN and VO_*x*_/SiO_2_ catalysts (Figure 1**)** are consistent with previous results.^34,37^ However, the origin of the high selectivity to C_3_H_6_/C_2_H_4_ and the inhibition of overoxidation to CO_*x*_ (especially to CO_2_) over BN have remained unclear so far and are the subject of this study.

### *Operando* PEPICO Spectroscopy

**Figure 2:**
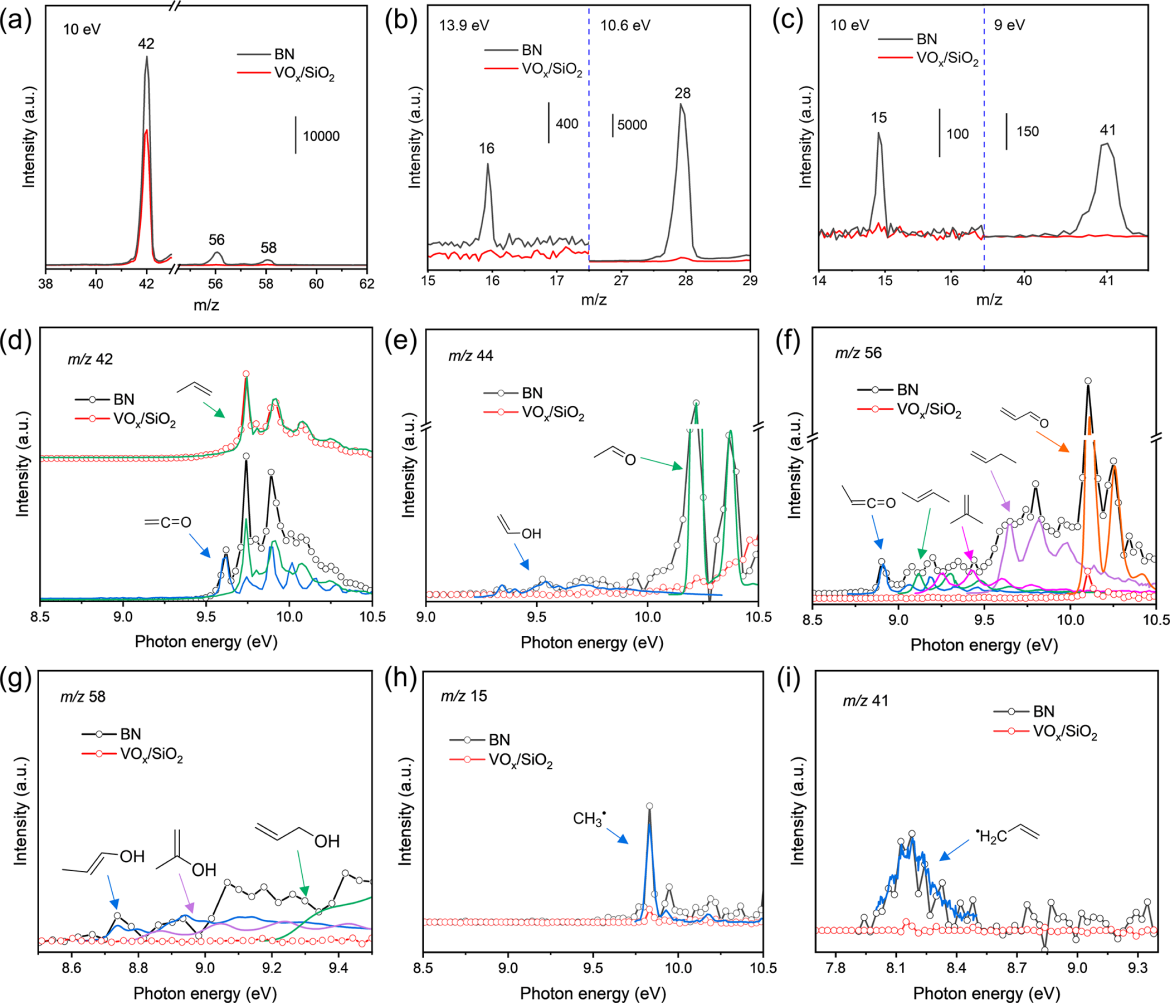
(a–c) ODHP photoionization mass spectra over BN and VO_*x*_/SiO_2_ at 600 °C at different photon energies; ms-TPES of (d) *m*/*z* 42, (e) *m*/*z* 44, (f) *m*/*z* 56, (g) *m*/*z* 58, (h) *m*/*z* 15, and (i) *m*/*z* 41 over BN and VO_*x*_/SiO_2_ at 600 °C; reaction conditions: 10 mg of catalyst, gas feed: 10% C_3_H_8_ and 20% O_2_ balanced in Ar; 10 mL/min total flow rate, 0.3 bar pressure; reference spectra are marked by arrows, see Table S1 for details.

*Operando* synchrotron PEPICO spectroscopy^38,39^ was utilized to detect stable products and elusive ODHP intermediates over BN and VO_*x*_/SiO_2_ catalysts. The PEPICO setup is described in Figure S1. In brief, a gas mixture of C_3_H_8_, O_2_, and Ar is fed into the preheated catalyst bed, and the continuous gas flow, with reactants, intermediates, and final products, expands from the reactor into high vacuum, forming a molecular beam. Molecular beam sampling freezes out the chemistry and suppresses quenching. The molecular beam travels through the skimmer and crosses the monochromatic vacuum ultraviolet beam in the ionization region. Soft photoionization yields photoelectrons and -ions, which are detected in delayed coincidence. Ion mass analysis yields photoionization mass spectra and, combined with electron kinetic energy analysis, allows us to plot photoion mass-selected threshold photoelectron spectra (ms-TPES) to identify the spectral carrier(s) of individual *m*/*z* peaks isomer-selectively.^40,41^ Mass spectra and ms-TPES are first compared at 600 °C based on a temperature-programmed surface reaction (TPSR) of adsorbed propane (C_3_H_8_) on BN from 500 to 700 °C (Figure S2). During ODHP operation, the *m*/*z* 42 peak dominates over both BN and VO_*x*_/SiO_2_, while the C_2_H_4_ (*m*/*z* 28) and CH_4_ (*m*/*z* 16) signals are much more intense over BN than over VO_*x*_/SiO_2_ (Figure 2a,b), consistent with the product distribution seen in Figure 1. A blank experiment without catalyst shows negligible product formation under the same conditions (Figure S3).

Moreover, small *m*/*z* 56 and 58 peaks are only visible over BN (Figure 2a). Based on the comparison of the *m*/*z* 42 ms-TPES with the propene and ethenone ketene reference spectra (Figure 2d), this peak can be assigned exclusively to C_3_H_6_ over VO_*x*_/SiO_2_ and to a mixture of C_3_H_6_ and C_2_H_2_O over BN. The ms-TPES of *m*/*z* 56 (Figure 2f) is assigned to a mixture of methylketene (C_3_H_4_O, CH_3_–CH=C=O), 2-propenal (C_3_H_4_O, CH_2_=CH–CH=O), and C_4_H_8_ (1-, 2-butene, and isobutene) over BN, based on reference and Franck–Condon simulated spectra. In contrast, only C_4_H_8_ is observed in Figure 1a as a final product. To the best of our knowledge, this is the first time that oxygenated species such as ketenes are observed as ODHP intermediates, likely because ketene and methylketene are unstable and evade detection by GC/MS even if present in the effluent.^42,43^ This illustrates the advantage of *operando* PEPICO in detecting elusive intermediates to provide mechanistic insights into catalytic mechanisms.^40^ The *m*/*z* 58 peak can be ascribed to propen-*m*-ol isomers (*m* = 1, 2, or 3, C_3_H_6_O) and propionaldehyde (Figures 2g and S4c), also newly observed in ODHP. Acetaldehyde and its high-energy tautomer, ethenol (vinyl alcohol, C_2_H_4_O, CH_2_=CH–OH), are detected over BN at *m*/*z* 44 (Figures 2e and S5). In addition to propionaldehyde and acetaldehyde, formaldehyde (HCHO) is also detected by its ms-TPES at *m*/*z* 30 (Figure S4b). The oxygenates (C_1–3_ aldehydes, C_2–3_ ketenes, and C_2–3_ enols) found over BN represent minor peaks compared to C_3_H_6_, C_2_H_4_, and CH_4_. However, the desorption of these intermediates from the catalyst surface, as implied by their detection, is crucial in preventing overoxidation (*vide infra*). Finally, the ion peaks at *m*/*z* 15 and *m*/*z* 41 detected over BN (Figure 2c) can be unambiguously assigned to methyl (CH_3_^•^) and allyl (C_3_H_5_^•^) radicals, respectively, based on the corresponding ms-TPES (Figure 2h and i).

**Figure 3:**
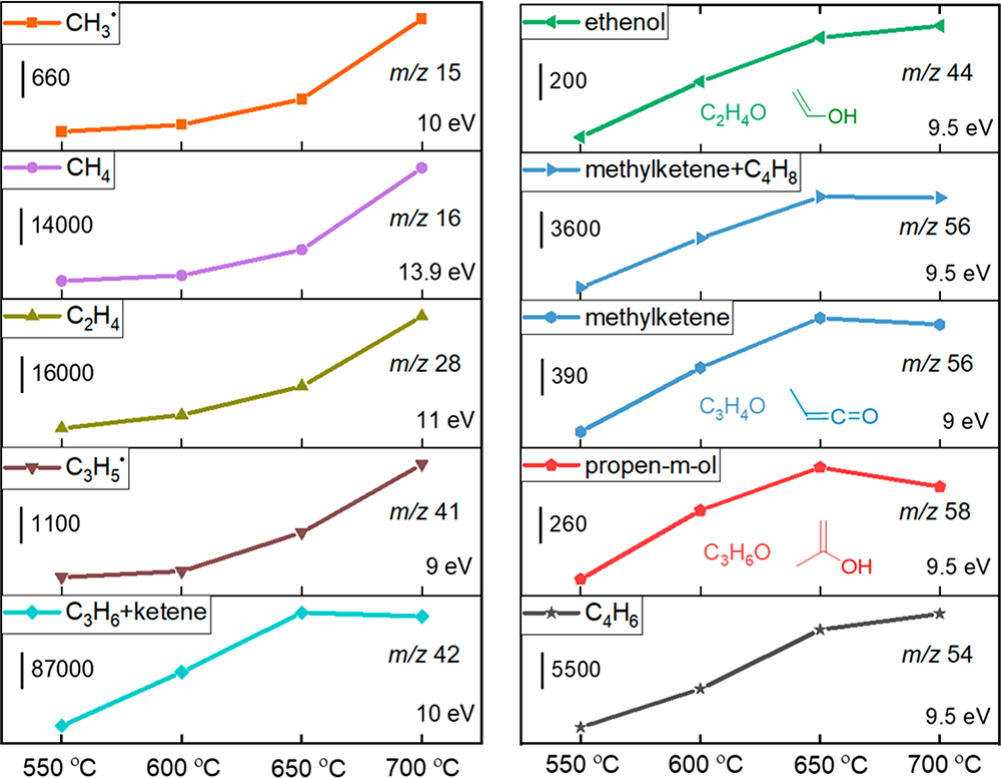
Photoionization mass spectrum peak integrals as a function of temperature in ODHP over BN; reaction conditions are the same as in Figure 2.

Motivated by the observation of oxygenates (aldehydes, ketenes, and enols) and radicals over BN, we further investigated the temperature-dependent product distribution over BN from 550 to 700 °C. The corresponding photoionization mass spectra are shown in Figure S6, while the integrated signals are presented in Figure 3. With increasing temperature, the CH_3_^•^ signal increases together with that of CH_4_, indicating that methane is likely formed by gas-phase H addition to CH_3_^•^ (see below). Since CH_3_^•^ is formed from C_3_H_8_, the observed C_2_H_4_ from the counter fragment C_2_H_5_ comes as no surprise. The C_3_H_5_^•^ signal appears at 600 °C (Figure S7) and continues to increase with temperature, suggesting increasing C_3_H_6_ decomposition at elevated temperatures. The rising C_3_H_8_ conversion and C_3_H_6_ activation affect the final C_3_H_6_ signal in opposite ways and result in constant propene abundance when the temperature is increased further from 650 to 700 °C (Figure 3). Ethenol, methylketene, and propen-*m*-ol signals all increase from 550 to 650 °C, but become stable or even decrease from 650 to 700 °C (Figure 3), implying that they may also be converted further to other products. The *m*/*z* 56 signal taken at a photon energy of 9.5 eV, which can be assigned primarily to C_4_H_8_ and, to a lesser degree, methylketene (Figures S8 and S9), also increases from 550 to 650 °C and finally becomes relatively stable. The simultaneous increase of the C_4_H_8_ signal together with CH_3_^•^ and C_3_H_5_^•^ from 550 to 650 °C suggests a possible gas-phase CH_3_^•^ + C_3_H_5_^•^ reaction to yield C_4_H_8_. The relatively stable *m*/*z* 56 signal between 650 and 700 °C is due to the simultaneous consumption of C_4_H_8_ by dehydrogenation, yielding C_4_H_6_ (*m*/*z* 54, Figure 3). CH_3_^•^ is produced by desorption from the catalyst surface after propane activation, because C_3_H_8_ cracking is not observed in the blank experiment (Figure S3). It is worth noting that C_2_ radicals, such as C_2_H_5_^•^, have not been observed. We consider the role of oxygenates in the ODHP mechanism and compare it with that in combustion. Notably, typical combustion temperatures are significantly higher than the ODHP reaction temperatures of 550–600 °C. However, analogous reactions are likely to occur on ODHP catalytically, since the catalyst provides a dehydrogenation active site.^44^ Vinyl alcohol (CH_2_=CH–OH) is less stable than its tautomer acetaldehyde^45^ by ca. 42 kJ mol^–1^ and yet plays a role in the partial oxidation of hydrocarbons and alcohols.^46^ The detection of C_2–3_ enols in this work implies a possible low-temperature surface-mediated enol formation side reaction in BN-catalyzed ODHP. In contrast to VO_*x*_/SiO_2_, full oxidation to CO_2_ is inhibited over BN, which is explained by only weakly surface-bound partially oxidized intermediates, which readily desorb into the gas phase and are detected. The observation of ketenes has mechanistic ramifications, as well. The presence of ethylketene (CH_3_CH_2_–CH=C=O) is evidenced by the *m*/*z* 70 ms-TPES (Figure S10) over BN. Ketenes have been widely reported as the key intermediates for olefin formation,^3,35,47,48^ which indicates an oxygenate-driven route in BN-catalyzed ODHP and will be addressed computationally in the following section.

### Computational Insights

**Figure 4:**
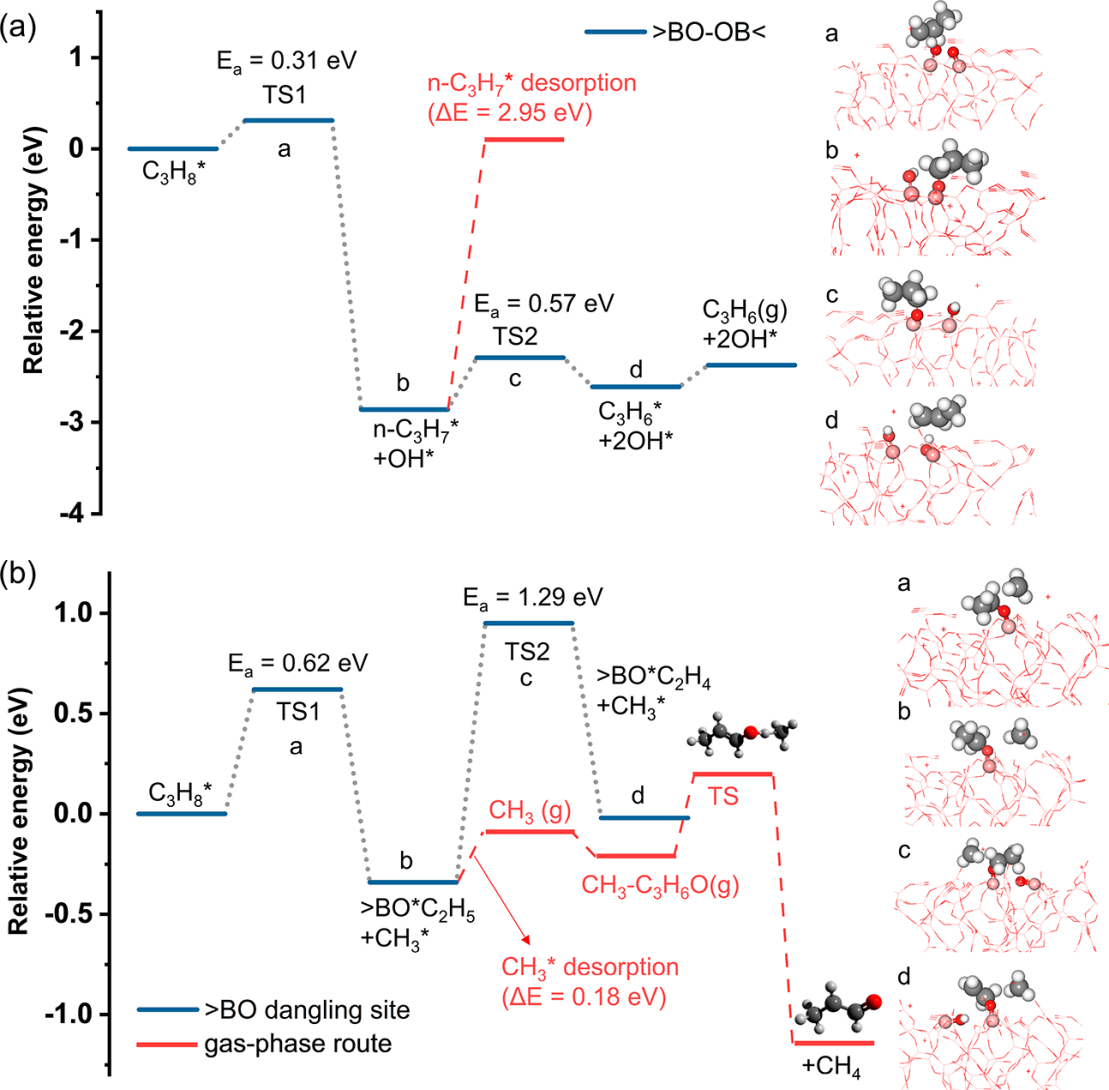
DFT-calculated energy profile for (a) C–H cleavage over the >BO–OB< site and (b) C–C cleavage over the >BO dangling site on the disordered B_2_O_3_ (101) surface. The corresponding minima and transition states are shown on the right.

Quantum chemical calculations were carried out for both surface-catalyzed and gas-phase reactions to reveal the origin of the reactive radicals and oxygenates detected over BN by *operando* PEPICO. Analogous theoretical calculations on ODHP over VO_*x*_-based catalysts have been carried out previously and, in light of propane overoxidation over VO*_x_*, are not discussed here further.^49−51^ Although a crystalline B_2_O_3_ (101) surface was used to simplify the BN active surface, it is accepted that the active catalyst surface corresponds more closely to amorphous boron oxide in ODHP.^34^ To simulate the real catalyst surface, *ab initio* molecular dynamics (AIMD) simulations were first carried out for the B_2_O_3_ (101) surface at the high temperatures of 1500–2000 K to achieve a disordered BO_*x*_ surface within the tractable time window of 2–4 ps (Figure S11). O_2_ chemisorption on BO_*x*_ forms peroxo-like >BO–OB< species preferentially, which is regarded as the main site for C_3_H_8_ activation.^28,31^ O_2_ chemisorption is modeled on the amorphous BO_*x*_ surface, and the adsorption configurations of C_3_H_8_ on the >BO–OB< site are optimized (Figure S12). After C–H cleavage at the >BO–OB< site (Figure 4a), C_3_H_8_* will transform into coadsorbed >BO–*n-*C_3_H_7_* and >BO–H* over a barrier of 0.31 eV (* represents adsorbed species). The formed *n-*C_3_H_7_* is strongly bound to the surface with nearly 3 eV desorption energy and will further dehydrogenate to yield C_3_H_6_* over a barrier of only 0.57 eV. Alternatively, C–C cleavage in C_3_H_8_ on >BO–OB< can also occur (Figure S13), but the cracked CH_3_* and C_2_H_5_* radicals are also strongly bound to the surface at desorption energies of 3.69 and 3.48 eV, respectively. This is much higher than the alternative C_2_H_4_ formation energy of 1.01 eV (Figure S14), which suggests that free radicals are not desorbed from the >BO–OB< site.

AIMD simulations of the BO*_x_* surface also yielded a >BO dangling site occurring at 1000 K (Figure S15). This site was also proposed to be active in boron-catalyzed ODHP.^31^ The C–C bond can be cleaved on the >BO dangling site (Figures 4b and S16). Here, C_3_H_8_* transforms into either >BO–C_2_H_5_*/CH_3_* or >BO–CH_3_*/C_2_H_5_*. The desorption energy of CH_3_* is only 0.18 eV from the former. For >BO–CH_3_*/C_2_H_5_*, calculations predict prompt C_2_H_5_* dehydrogenation to C_2_H_4_* (Figure S17), implying that C_2_H_5_* is unlikely to desorb into the gas phase. Moreover, C_3_H_6_ can also be activated on the >BO dangling site, readily forming gas-phase C_3_H_5_^•^ with a desorption energy of 0.33 eV (Figure S18). These results identify the >BO dangling site as the source for the experimentally observed CH_3_^•^ and C_3_H_5_^•^ radicals. In addition to the C–C cleavage route on the >BO dangling site, we also found a comparable C–H cleavage barrier toward *n-*C_3_H_7_* and *i-*C_3_H_7_* (Figure S19). The adsorbed *i-*C_3_H_7_* intermediate will spontaneously transform into C_3_H_6_ by terminal H abstraction (Figure S20). In contrast, *n-*C_3_H_7_* may either transform into C_3_H_6_ over a barrier of 0.17 eV or propagate into the gas phase with a desorption energy of only 0.39 eV (Figure S21). Despite surface-catalyzed *n-*C_3_H_7_* to C_3_H_6_ conversion having a lower energy barrier, *n-*C_3_H_7_* desorption may effectively compete, especially at high temperatures. The propyl radical intermediate was, however, not detected experimentally in the gas phase, in contrast to CH_3_^•^ and C_3_H_5_^•^. We first have to consider gas-phase radical chemistry to conclude whether this indicates nondesorption or a short gaseous lifetime of *n-*C_3_H_7_.

In addition to radicals, gas-phase reactive oxygenates are also detected by *operando* PEPICO. As guided by previous gas-phase mechanistic insights on, for example, the role of enols in combustion,^44^ we applied the G4 composite method to address possible gas-phase reaction pathways. The formation of enols and ketenes may proceed similarly to a combustion chemistry pathway. Enols, for instance, are likely produced via partial hydrocarbon oxidation on the catalyst surface in ODHP, followed by desorption into the gas phase. In the gas phase, the allyl + O_2_ reaction affords formaldehyde (observed in Figure S4) and the H_2_C–C(=O)H radical, in an exothermic reaction (−2.75 eV) after an entrance barrier of 0.55 eV,^52^ which is likely reduced in the presence of BN. The H_2_C–C(=O)H radical can either be dehydrogenated to yield ketene or hydrogenated to vinyl alcohol or acetaldehyde (*m*/*z* 44, Figures S5, S8), which were all observed in this study. On the one hand, we found computationally that C_2–3_ enols (C_2_H_4_O, C_3_H_6_O) can be converted to C_2–3_ ketenes (C_2_H_2_O, C_3_H_4_O) via dehydrogenation by, for example, a CH_3_^•^ radical over a low energy barrier of 0.46 eV for C_2_H_2_O and 0.40 eV for C_3_H_4_O (Figure S22). This H abstraction step of propen-1-ol by CH_3_^•^ after CH_3_^•^ desorption is included in Figure 4b for comparison. Since computational results do not rule out *n-*C_3_H_7_^•^ desorption from the surface, we also investigate H abstraction from enols via *n*C_3_H_7_^•^, which was found to be associated with a similar energy barrier to that with CH_3_^•^ radicals (Figure S23) and could contribute to quenching *n-*C_3_H_7_^•^ by C_3_H_8_ formation. Hydrogen abstraction from C_2–3_ enols by the resonantly stabilized C_3_H_5_^•^ involves a high-energy transition state at ∼0.8 eV (Figure S24); that is, the allyl radical is unlikely to participate in the first hydrogen transfer from enols. However, the second H-transfer step, to finally yield ketenes, is computed to be downhill, irrespective of the H-acceptor free radical. These observations imply that enols can easily dehydrogenate into ketenes in the presence of the H-acceptor radical species CH_3_^•^, *n-*C_3_H_7_^•^, or—as far as the second H-transfer step is concerned—C_3_H_5_^•^. However, *n-*C_3_H_7_^•^ radicals are also H-donor agents. In fact, a barrier-free H-transfer reaction is predicted once an *n-*C_3_H_7_^•^ radical collides with CH_3_^•^, C_3_H_5_^•^, or another *n-*C_3_H_7_^•^ associated with the release of 2 to 3 eV of energy (Figure S25), contributing to rapid quenching of *n-*C_3_H_7_^•^ by H loss to C_3_H_6_ and the associated formation of CH_4_, C_3_H_6_, or C_3_H_8_. At a 0.3 bar reactor pressure, the gas-phase collision frequency is on the order of 10^8^ s^–1^ at 600 °C, which implies prompt quenching of the *n-*C_3_H_7_^•^ radical in the presence of H acceptors. Therefore, the absence of *n-*C_3_H_7_^•^ radical detection can be ascribed (1) to the more favorable surface-confined *n-*C_3_H_7_* to C_3_H_6_ reaction path compared to *n-*C_3_H_7_* desorption as well as (2) to its low expected lifetime in the gas phase under the reaction conditions and in the presence of H-acceptors. Since ketene and methyl radicals are both observed in the gas phase, and surface methyl and ethyl species are predicted (Figure S13), ethylketene may also be formed by the methylation of methylketene or ethylation of ketene. The formation of ethylketene is proven by *operando* PEPICO (Figure S10). Methylketene and ethylketene are known as precursors for C_2_H_4_ and C_3_H_6_ via decarbonylation.^35^ However, these processes can only take place over a high barrier (>3 eV) in the gas phase (Figure S26). Therefore, ketene decarbonylation is surface catalyzed and proceeds over BN to form CO and olefins, both of which desorb from the surface easily. This also explains how CO is formed in the ODHP over BN. Additionally, the detection of gas-phase oxygenates only over BN suggests they are prone to desorb from the BN surface, which also prevents deep oxidation to CO_2_.

### Reaction Mechanism

**Figure 5:**
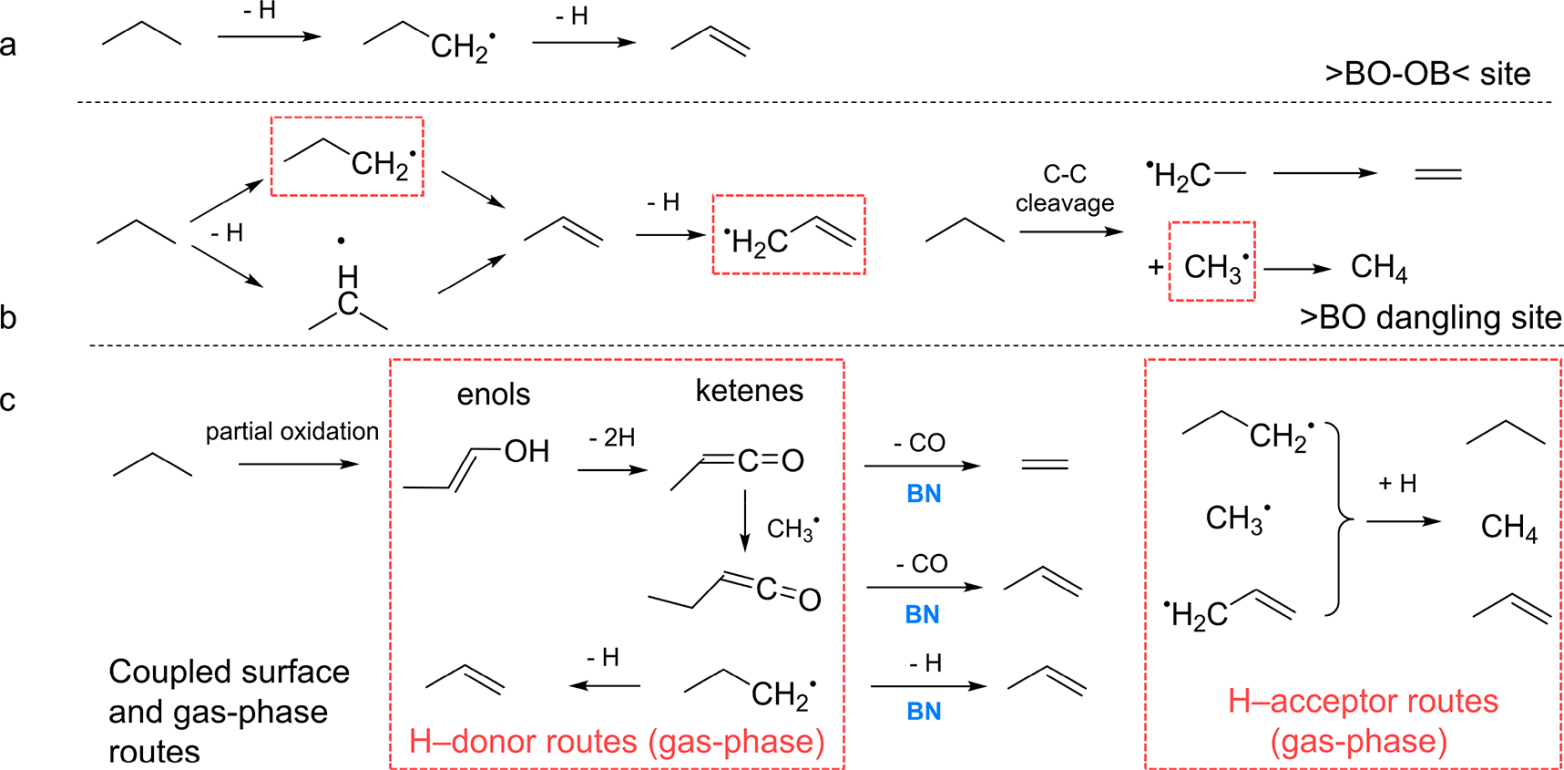
ODHP mechanism over the BN catalyst, including coupled surface-confined and gas-phase reaction routes.

By combining *operando* PEPICO experiments and quantum chemical calculations, we have formulated a comprehensive ODHP mechanism over BN in Figure 5, involving coupled surface-confined and gas-phase reactions. Gas-phase radicals and reactive oxygenates are detected by *operando* PEPICO. Calculations predict that C_3_H_8_ transforms into *n-*C_3_H_7_* on the >BO–OB< site (Figure 5a), which only desorbs as C_3_H_6_ after further dehydrogenation. C_3_H_8_ can also dissociate to CH_3_* and C_2_H_5_*. However, both are strongly surface-bound and do not desorb from the >BO–OB< site. On the >BO dangling site, *n-*C_3_H_7_* and *i-*C_3_H_7_* are both formed after C–H activation (Figure 5b). However, *i-*C_3_H_7_* yields C_3_H_6_ instantaneously, and only *n-*C_3_H_7_* may desorb due to its low desorption energy of 0.39 eV. Yet, *n-*C_3_H_7_^•^ is not detected in the gas-phase experimentally, which can be attributed to the more favorable surface reaction to C_3_H_6_ and its short gas-phase lifetime in the presence of H acceptors. Additionally, >BO–C_3_H_6_* can be activated to form C_3_H_5_* on the >BO dangling site, followed by easy desorption to form the allyl free radical. C–C cleavage on the >BO dangling site has two different reaction routes, forming >BO–C_2_H_5_*/CH_3_* or >BO–CH_3_*/C_2_H_5_*. The former yields gas-phase CH_3_^•^, and the latter will transform to C_2_H_4_. Thus, free radicals detected by *operando* PEPICO spectroscopy are derived from the >BO dangling site. Partial oxidation products can also be observed in the gas phase with BN as a catalyst (Figure 5c). The desorption of oxygenates from the catalyst surface indicates weak bonding, which is crucial in preventing catalytic oxygenation of oxygenates into CO and CO_2_.

After the desorption of radicals and oxygenates, gas-phase H-abstraction chemistry will be initiated (Figure 5c). Aside from the self-reaction to form C_2_H_6_, the CH_3_^•^ free radical can also abstract an H from enols. Gas-phase dehydrogenation into ketenes with H-acceptors (e*.*g., CH_3_^•^, *n-*C_3_H_7_^•^, or, in the second step, C_3_H_5_^•^) can take place readily based on G4 calculations (Figures S22–S24). Meanwhile, CH_3_^•^, *n-*C_3_H_7_^•^, and C_3_H_5_^•^ will form CH_4_, C_3_H_8_, and C_3_H_6_ after abstracting an H. The formed methylketene and ethylketene can then undergo surface-assisted decarbonylation to form C_2_H_4_ and C_3_H_6_ (Figure 5c). This new C_3_H_6_ and C_2_H_4_ formation mechanism is driven by methyl- and ethylketene in BN-catalyzed ODHP, which is observed for the first time. In addition to surface-bound propyl radicals (C_3_H_7_*) previously considered to be the only intermediate in C_3_H_6_ formation, this establishes partially surface-oxidized oxygenates such as C_3_ enols (propen-1-ol, C_3_H_6_O) as precursors to C_3_H_6_ via a gas-phase reaction sequence including dehydrogenation, methylation, and subsequent surface-confined ethylketene decarbonylation over the BN catalyst (Figure 5c). C_4_H_8_, only observed over a BN catalyst, can be ascribed to the gas-phase CH_3_^•^ + C_3_H_5_^•^ association reaction. Importantly, oxygenates desorb easily from the BN surface before deep oxidation to CO_2_, and ketene decarbonylation is the main CO source over BN. This explains why carbon monoxide is the main deeply oxidized byproduct over BN with negligible CO_2_ formation. In contrast, CO_2_ is dominantly formed over vanadium-based catalysts, and no oxygenates can be observed in the gas phase, due to strong binding to the surface and rapid catalytic deep oxidation. Additionally, quantum chemical calculations indicate that *n-*C_3_H_7_^•^ could escape from the catalyst surface. If so, *n-*C_3_H_7_^•^ will participate in gaseous reactions with H-donor or H-acceptor species to form C_3_H_6_ or C_3_H_8_ (Figure 5c). In this coupled surface and gas-phase route (Figure 5c), H-acceptor radicals and H-donor oxygenates (or *n-*C_3_H_7_^•^) react via fast H migration. The selectivity to CO rises with temperature (Figure 1a), which suggests a competitive advantage of the oxygenate route at high temperatures. Although CO is an undesirable ODHP product, it is still more useful than CO_2_, making the transformation of C_3_H_8_ and O_2_ to olefins and CO over boron-based catalysts alluring. Calculations also revealed the different role of chemistry at the >BO–OB< main and the >BO dangling sites. Such insights help tune the catalytic activity in a targeted way and may guide the rational design of boron-based catalysts with higher selectivity.

## Conclusions

In addition to propyl as the main precursor of propene, we identify a new H-acceptor radical- and H-donor oxygenate-driven propene formation route by *operando* PEPICO spectroscopy. Partially oxidized enols dehydrogenate in the presence of H-acceptor radicals to ketenes, which then transform to olefins by decarbonylation. Ethylketene is the observed precursor of propene in this route, which is unique to BN and absent over a vanadium-based catalyst. Moreover, we report that free radicals are derived solely from the >BO dangling site, as they are strongly bound at the >BO–OB< main site. These results not only help explain the ever-elusive ODHP reaction mechanism on BN but also aid to design next-generation boron-based catalysts for enhanced ODHP performance.

## Data Availability

Data presented in the main figures of the manuscript and Supporting Information are publicly available through the repository: 10.16907/a8b0a8e7-8784-4789-847f-d21e52d4334c.

## Supporting Information Available

The experimental methods, detailed PEPICO, and theoretical calculation data (PDF)
