# Supplementary material for: Unraveling Radical and Oxygenate Routes in the Oxidative Dehydrogenation of Propane over Boron Nitride
Source: J Am Chem Soc. 2023 Mar 3;145(14):7910–7. doi: 10.1021/jacs.2c12970 (PMC10103127; doi:10.1021/jacs.2c12970)
Supplement: Supplementary file 1 — ja2c12970_si_001.pdf [file ja2c12970_si_001.pdf]

Supporting Information

Unraveling Radical and Oxygenate Routes in the Oxidative Dehydrogenation of Propane over Boron Nitride

Zihao Zhang,[1†] Jinshu Tian,[2†] Xiangkun Wu,[1] Ivan Surin,[3] Javier Pérez-Ramírez,[3,*] Patrick Hemberger,[1,*] Andras Bodi,[1,*]

[1] Paul Scherrer Institute, 5232 Villigen, Switzerland

[2] College of Chemical Engineering, Zhejiang University of Technology, Hangzhou 310014, China

[3] Institute for Chemical and Bioengineering, Department of Chemistry and Applied Biosciences, ETH Zurich, Vladimir-Prelog-Weg 1, 8093 Zurich, Switzerland

*Corresponding authors. Email: jpr@chem.ethz.ch; patrick.hemberger@psi.ch; andras.boedi@psi.ch

†These authors contributed equally

Experimental Methods

VO_x/SiO_2 and BN synthesis and preparation. 10 wt% VO_x/SiO_2 catalyst was synthesized via a wet impregnation method.[1] 293 mg NH_4VO_3 (99%) of was dissolved in 60 mL DI water. The resulting aqueous solution was added drop-wise to 1 g SiO_2 (99.7 %, 100 mesh) followed by stirring for 1 h and drying at 110 °C overnight. The prepared precursor was calcined at 600 °C in static air for 6 h to yield the VO_x/SiO_2 catalyst. Commercial BN was purchased from Strem Chemicals, Inc. (hexagonal crystalline solid, 99%). Prior to use, BN was pretreated at 600 °C in 10% C_3H_8 and 20% O_2 balanced in Ar for 6 h.

Catalytic evaluation. ODHP was evaluated at atmospheric pressure in a continuous-flow fixed-bed microreactor (Fig. S27). The gases C_3H_8 (Linde, purity 3.5), O_2 (PanGas, purity 5.0), Ar (PanGas, purity 5.0, internal standard), and He (PanGas, purity 5.0, carrier gas), were fed using digital mass-flow controllers (Bronkhorst) to the mixing unit, equipped with a pressure indicator. A quartz microreactor of 10 mm inner diameter was loaded with the catalyst (W_{cat} = 0.02 g, diluted with 0.08 g of SiC, particle size 0.2–0.4 mm) and placed in a homemade electrical oven. A Type K thermocouple, fixed in a coaxial quartz thermowell with the tip positioned in the center of the catalyst bed, was used to control the temperature during the reaction. Prior to testing, the catalyst was heated in a He flow to the desired bed temperature (T_{bed} = 500–700 °C) and then allowed to stabilize for at least 2 h while the reaction mixture (10 vol.% C_3H_8, 20 vol.% O_2, and 4 vol.% Ar) was fed at a total volumetric flow of F_T = 66 cm^3 min^{-1}. Carbon-containing compounds (C_3H_8, C_3H_6, C_2H_6, C_2H_4, CH_4, CO, CO_2, and C_4H_8) and Ar were quantified on-line *via* a gas chromatograph equipped with a GS-Carbon PLOT column and coupled to a mass spectrometer (GCMS, Agilent, GC 7890B, Agilent MSD 5977A). Four data points were acquired at 15 min intervals over the course of 1 h at each temperature. If steady-state performance was reached, the arithmetic average of the first and the last data points are used to obtain the catalytic performance metrics, *i.e.*, conversion and selectivity.
The conversion of C_3H_8 was calculated according to Equation (1),

$$X_{i},\% = \frac{n_{C_3H_8}^{inlet} - n_{C_3H_8}^{outlet}}{n_{C_3H_8}^{inlet}} \cdot 100 \qquad (1)$$

where $n_{C_3H_8}^{inlet}$ and $n_{C_3H_8}^{outlet}$ denote the molar flows of C_3H_8 at the reactor inlet and outlet, respectively. Selectivity towards individual products was determined according to Equation (2),

$$S_{i},\% = \frac{n_{i}^{outlet} \cdot \frac{v_i}{3}}{n_{C_3H_8}^{inlet} \cdot X_{C_3H_8}} \cdot 100 \qquad (2)$$

where v_i is the number of C atoms in the product molecule (*i.e.*, v = 4 for C_4H_8, v = 3 for C_3H_6 etc.).

***Operando* PEPICO measurements.** PEPICO experiments were carried out at the VUV beamline of the Swiss Light Source by using the double imaging Photoelectron Photoion Coincidence (CRF-PEPICO) spectrometer.[2] The PEPICO setup is shown in Fig. S1, and the detailed description of the beamline[3] and the endstation can be found elsewhere.[4] 10 mg of catalyst is packed in the quartz microreactor (inlet, 3 mm inner diameter; outlet, 2 mm inner diameter) held in place with quartz wool at both ends. 10% C_3H_8 and 20% O_2 balanced in Ar with a total flow rate of 10 mL/min is then fed to the heated reactor. The typical pressure in the reactor inlet is 300 mbar. After reaction, the effluent leaves the reactor and forms a molecular beam in the source chamber at a pressure of

5×10^{-5} mbar, providing a low-collision environment and preventing the reactive species from being quenched. The molecular beam is skimmed and travels towards the ionization chamber (10^{-6} mbar), followed by ionization with monochromatic VUV synchrotron radiation. For photon energies higher than 10.6 eV, radiation at higher grating orders are suppressed in the differentially pumped rare gas filter filled with 1 kPa of an Ar, Ne and Kr mixture. For the photon energy lower than 10.6 eV, higher grating orders are suppressed by an MgF_2 window. The generated photoelectrons and -ions are extracted in opposite direction by a constant 216 V cm^{-1} electric field. Both are detected in velocity map imaging conditions by position-sensitive delay-line anode detectors (Roentdek, DLD40). Photoionization mass spectra are obtained by detecting electrons and ions in delayed coincidence. Photoion mass-selected threshold photoelectron spectra (ms-TPES) are obtained by selecting close-to zero kinetic energy (threshold) energies[5] and plotting the coincident ion signal in an *m*/*z* channel as a function of photon energy. Isobaric and isomeric spectral carriers are discerned by comparing ms-TPES with known reference spectra or Franck–Condon simulations. Temperature-programmed surface reaction (TPSR) was also carried out utilizing the PEPICO setup. Briefly, the catalyst was pretreated in C_3H_8 at 500 °C for 30 mins, then purged with Ar for 20 mins. Thereafter, the catalyst was heated up to 700 °C in O_2 with 10 °C/min ramping rate, and while collecting mass spectra.

Calculations. Periodic DFT calculations were carried out using the CP2K package.[6] The generalized-gradient approximation with the spin-polarized Perdew–Burke–Ernzerhof functional was utilized for the exchange–correlation energy.[7] The valence electron wave function was expanded in an optimized double-ζ Gaussian basis set with an auxiliary plane wave basis set and a cutoff energy of 350 Ry. Core electrons were represented with scalar relativistic norm-conserving pseudopotentials with 3, 6, 1 and 4 valence electrons for B, O, H and C, respectively.[8] Brillouin zone integration were performed with a reciprocal space mesh consisting of only the gamma point. To account for short-range van der Waals dispersion interactions, the DFT-D3 scheme[9] with an empirical damped potential term was included into the electronic energy. The convergence criterion for the maximum force is set as 4.5×10^{-4} au. B_2O_3 (oxidized boron) is selected as the surface representative of BN catalyst during ODHP reaction condition is based on previous research.[10,11] The geometry optimization of crystalline B_2O_3-I (4×4×2) was performed using the Broyden–Fletcher–Goldfarb–Shanno (BFGS) algorithm, relaxing both the lattice parameters and atomic coordinates. The optimized lattice parameters of crystalline B_2O_3-I were $a = b = 4.36$ Å and $c = 8.51$ Å, in good agreement with the experimental values ($a = b = 4.34$ Å, and $c = 8.34$ Å with $\alpha = \beta = 90^o$, $\gamma = 120°$).[12] The B_2O_3 (101) facet was designed by cleaving the B_2O_3-I crystal in the (101) direction. The constructed B_2O_3 (101) facet consists of 240 (96 B + 144 O) atoms with lattice parameters at $a = 19.7$ Å, $b = 17.61$Å, and $c = 25$ Å ($\alpha = \beta = 90^o$, $\gamma = 116.6°$). The climbing image nudged-elastic-band method (CI-NEB) including five replicas was employed to determine the transition states (TS) for the elementary reactions.[13] The TS was denoted by the highest image along the minimum energy path. The energy barrier (E_a) of each elementary reaction was calculated by the energy difference between the TS and the initial state neglecting the change in zero-point energy. Ab initio molecular dynamics (AIMD) simulations were carried out by sampling the canonical ensemble using Nose–Hoover thermostats with a time step of 0.5 fs at a finite temperature.[14] AIMD was carried out for about 2 ps at a temperature of 2000 K and 1500 K starting from B_2O_3 (101) surface. The above run temperature was selected to be high to make the system reach a completely disordered state within the 2 ps. Afterwards, the system temperature was gradually decreased from 1500 K to the final target temperature of 850 K by running for another 3 ps. Gas-phase quantum chemical calculations were performed using Gaussian 16.[15]

Optimized geometries for reactants, transition states, intermediates and products were obtained using density functional theory at the B3LYP/6–311++G(d,p) level. Transition states were found using constrained geometry scans and stationary points were checked by frequency analysis. The relevant stationary points on the potential energy surface were re-calculated using the G4 composite method to obtain activation energies.[16]

Supplementary Tables

Table S1 Source of ms-TPES for the species of interest

Species	Molecular Structure	Reference
Ketene (*m*/*z* 42, C_2H_2O)	=C=O	[17]
Methylketene (*m*/*z* 56, C_3H_4O)	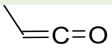	[18]
Propylene (*m*/*z* 42, C_3H_6)	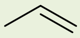	measured in-house
Butene (*m*/*z* 56, C_4H_8)	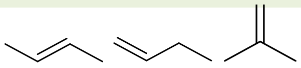	[19]
1,3-butadiene (*m*/*z* 54, C_4H_6)	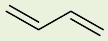	[20]
Ethenol (*m*/*z* 44, C_2H_4O)	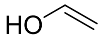	FC simulations
Propen-1-ol (*m*/*z* 58, C_3H_6O)	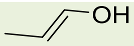	FC simulations
Propen-2-ol (*m*/*z* 58, C_3H_6O)	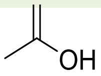	FC simulations
Propen-3-ol (*m*/*z* 58, C_3H_6O)	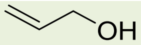	FC simulations
2-propenal (*m*/*z* 56, C_3H_4O)	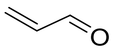	FC simulations
2-Butene, 2-methyl- (*m*/*z* 70, C_5H_{10})	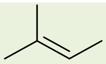	FC simulations
Dimethylketene (*m*/*z* 70, C_4H_6O)	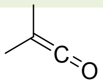	FC simulations
Ethylketene (*m*/*z* 70, C_4H_6O)	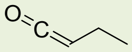	FC simulations
Acetone (*m*/*z* 58, C_3H_6O)	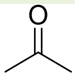	measured in-house
Acetaldehyde (*m*/*z* 44, C_2H_4O)	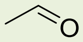	FC simulations
Propionaldehyde (*m*/*z* 58, C_3H_6O)	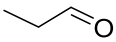	FC simulations
Ally radical (*m*/*z* 41, C_3H_5)	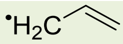	measured in-house[21]
Methyl radical (*m*/*z* 15, CH_3)	$CH_3^{\bullet}$	[22]
Formaldehyde (*m*/*z* 30, HCHO)	=O	measured in-house

Franck–Condon (FC) simulations: The double harmonic simulations used density functional theory at the B3LYP/6–311++G(d,p) level at a temperature of 500 K and were convoluted with a Gaussian of 150 cm^{-1} full width at half maximum (FWHM) to account for the rotational envelope.

Table S2 Ionization energies of *m*/*z* 70 molecules taken from the NIST Chemistry Webbook.[23]

Name	Chemical Structure	ionization energy
2-methyl-2-butene	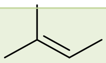	8.69
1-Pentene	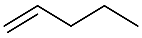	9.49
E-2-Pentene	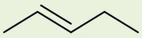	9.04
Z-2-Pentene	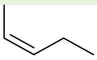	9.01
3-Methyl-1-butene	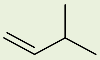	9.52
2-Methyl-1-butene	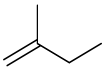	9.12
Cyclopentane	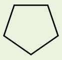	10.33
Dimethylketene	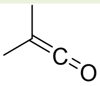	8.38
Ethylketene	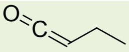	8.8

Supplementary Figures

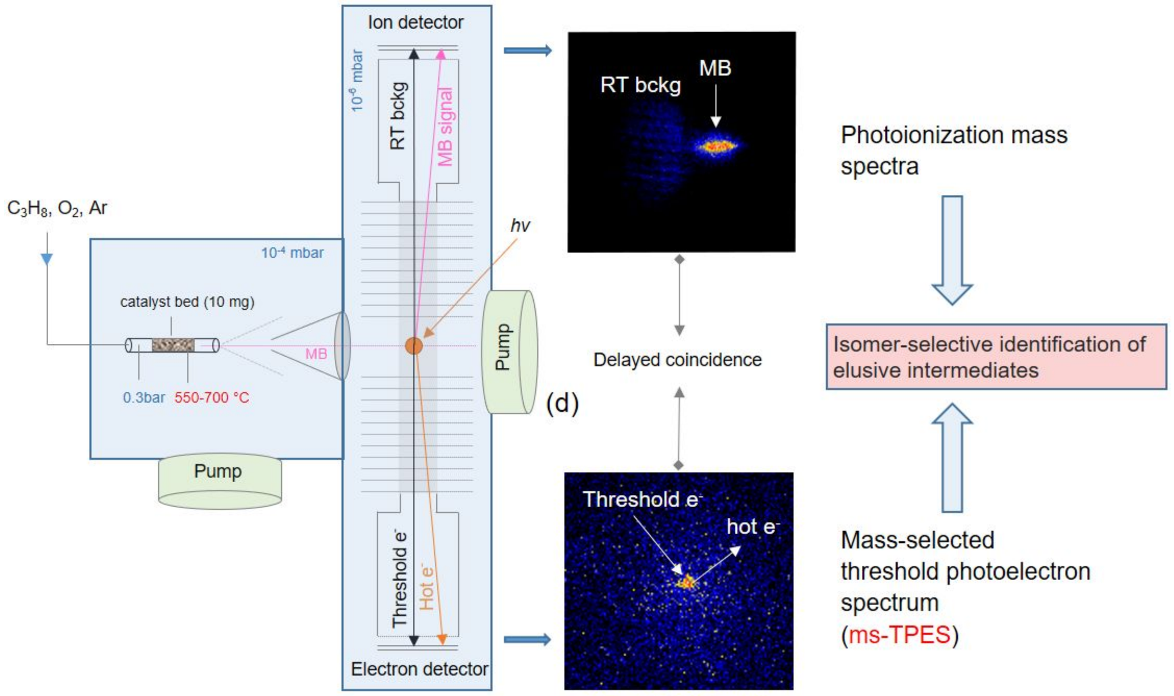

Fig. S1 PEPICO setup probing ODHP. The gas mixture is fed to the quartz tube microreactor placed in the source chamber on the left. 10 mg of catalyst is packed in the heated microreactor. The intermediates and products desorbed from the catalyst surface expand from the reactor into vacuum and form a molecular beam. The beam is skimmed as it enters the ionization chamber and proceeds towards the ionization region. The gaseous sample is photoionized by monochromatic synchrotron radiation. Photoions and photoelectron are extracted in opposite directions and detected in delayed coincidence. Photoionization mass spectra, photoionization spectra, and photoion mass-selected threshold photoelectron spectra (ms-TPES) are obtained to identify the spectral carriers isobar- and isomer-selectively.

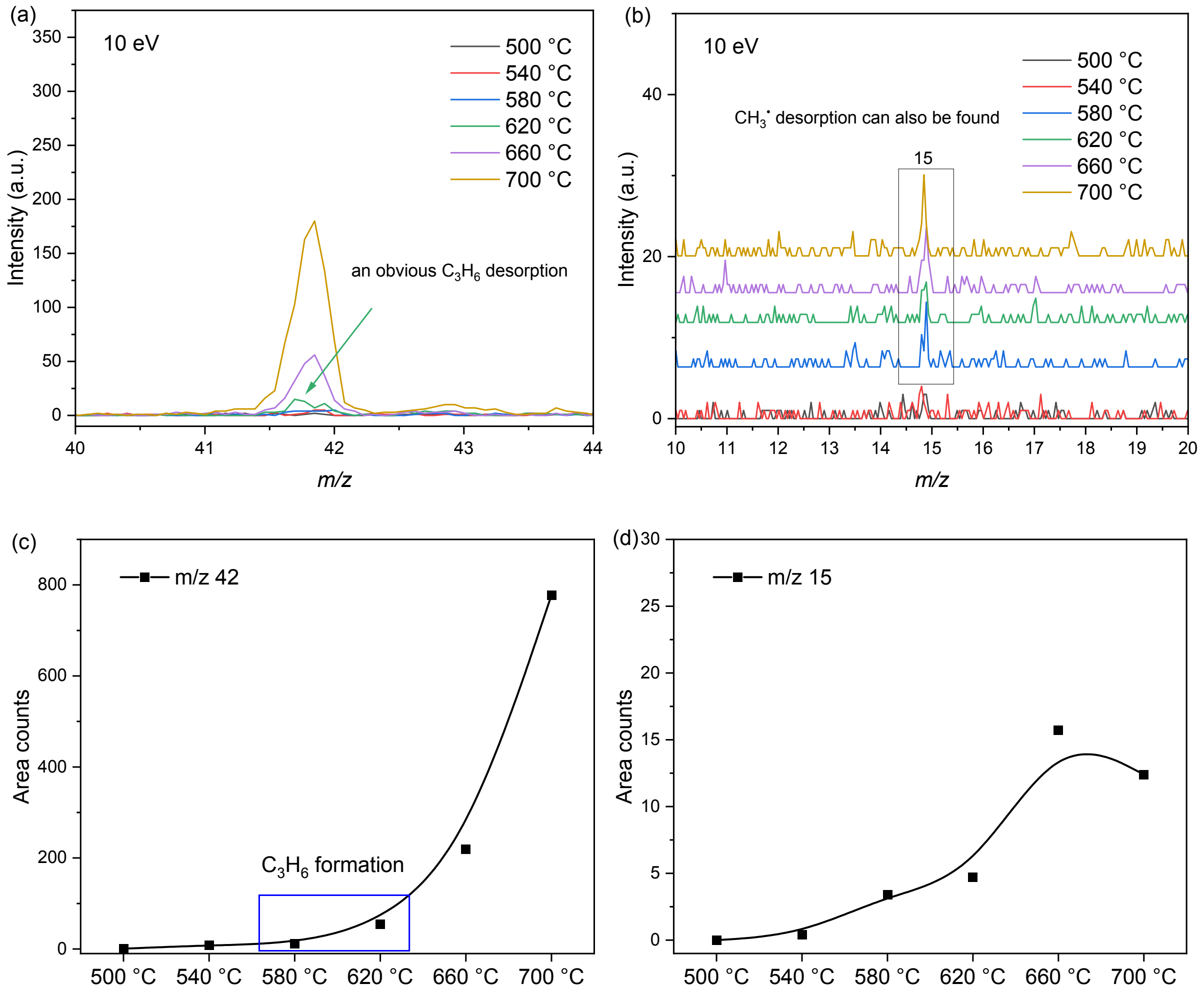

Fig. S2 Temperature-programmed surface reaction (TPSR) of adsorbed propane (C_3H_8) on BN from 500 to 700 °C; (a,b) photoionization mass spectra at 10.0 eV; peak areas of (c) *m*/*z* 42 (dominantly C_3H_6), (d) *m*/*z* 15 ($CH_3^{\bullet}$) as a function of temperature. Based on steady-state ms-TPES scans, ketene is expected to be at most a minor contributor to the *m*/*z* 42 peak. $CH_3^{\bullet}$ and C_3H_6 formation sets in at a similar temperature of ~600 °C. Conditions: Catalyst was pretreated in C_3H_8 at 500 °C for 30 min, then purged with Ar for 20 min. Thereafter, the catalyst was heated up to 700 °C in O_2 with 10 °C/min ramp rate, while collecting mass spectra.

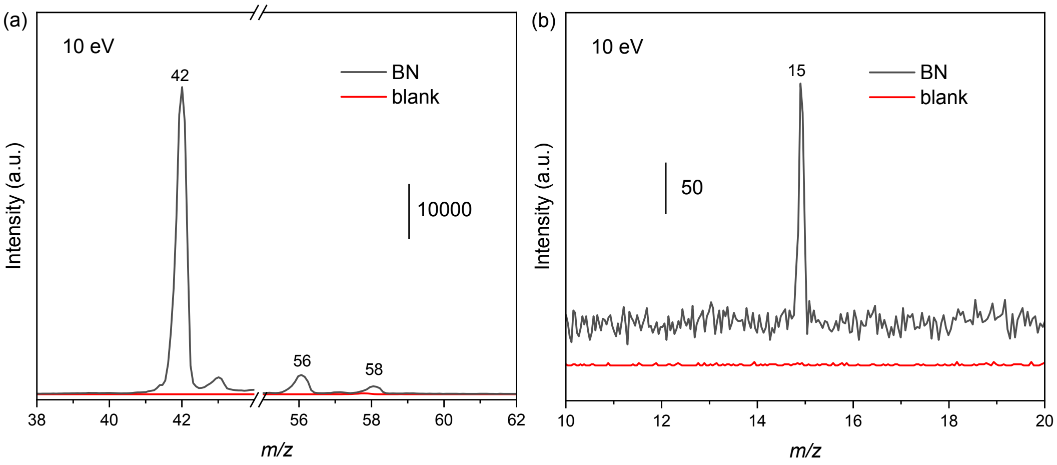

Fig. S3 Comparison of ODHP photoionization mass spectra over BN and in a blank experiment at 600 °C, recorded at a photon energy of 10 eV.

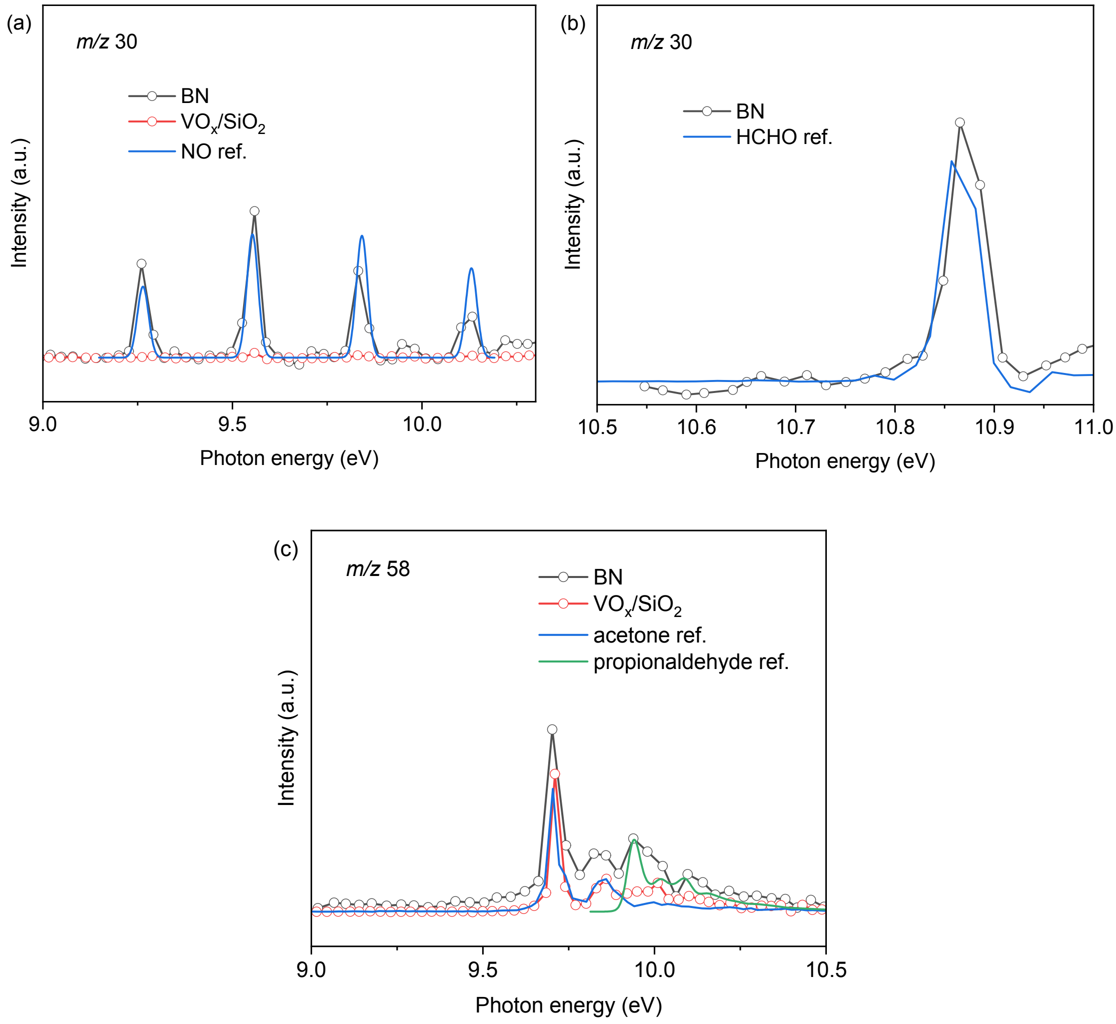

Fig. S4 ms-TPES of (a,b) *m*/*z* 30 and (c) *m*/*z* 58 in ODHP over BN and VO_x/SiO_2 at 600 °C.

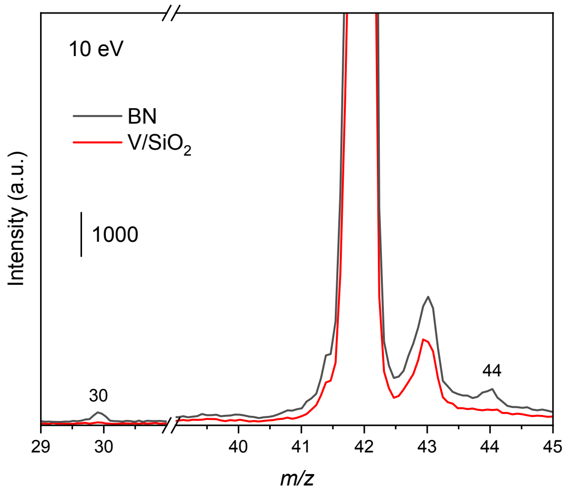

Fig. S5 ODHP photoionization mass spectra over BN and VO_x/SiO_2 at 600 °C recorded at 10 eV photon energy. Small *m*/*z* 30 and *m*/*z* 44 peaks are also observed over BN. The *m*/*z* 30 peak is assigned to NO here, because of the higher formaldehyde ionization energy (Fig. S4b). The *m*/*z* 44 peak is mainly ethenol (vinyl alcohol) since acetaldehyde has higher ionization energy (Fig. 2e).

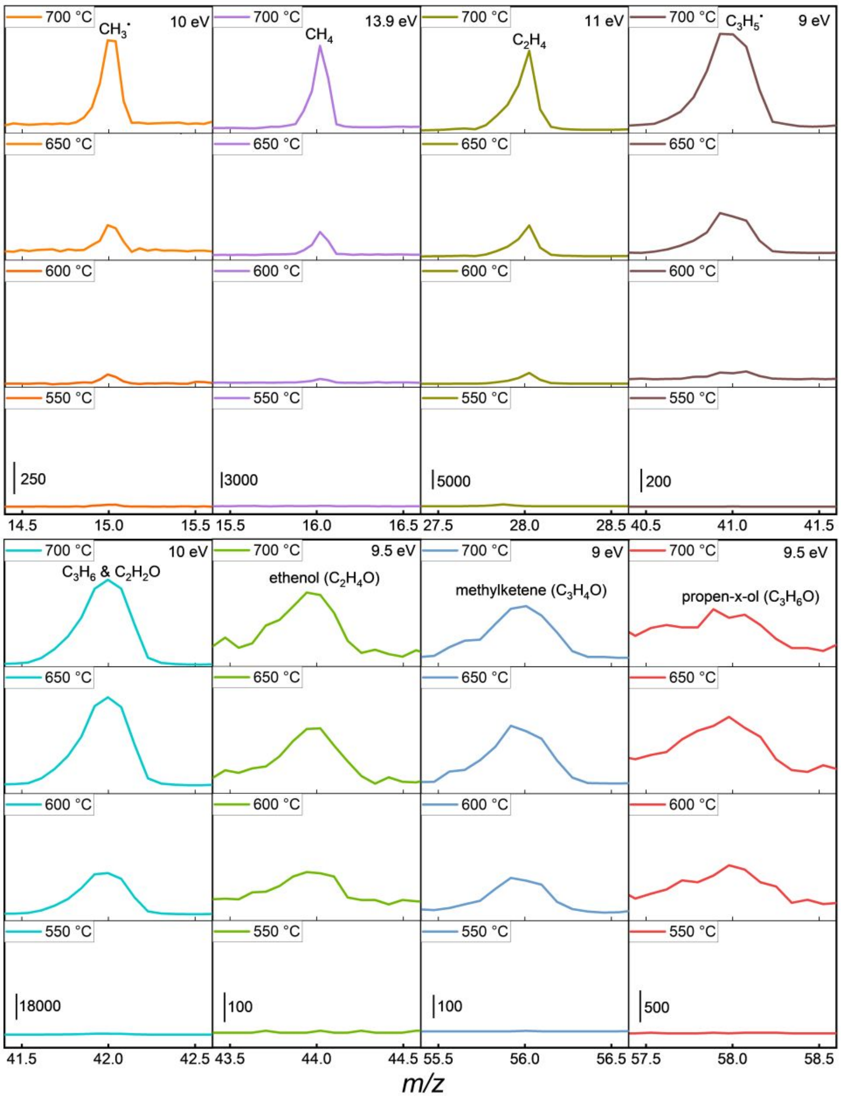

Fig. S6 Photoionization mass spectra of different stable and unstable species as a function of temperature in the ODHP reactions over BN; Reaction conditions as in Fig. 2.

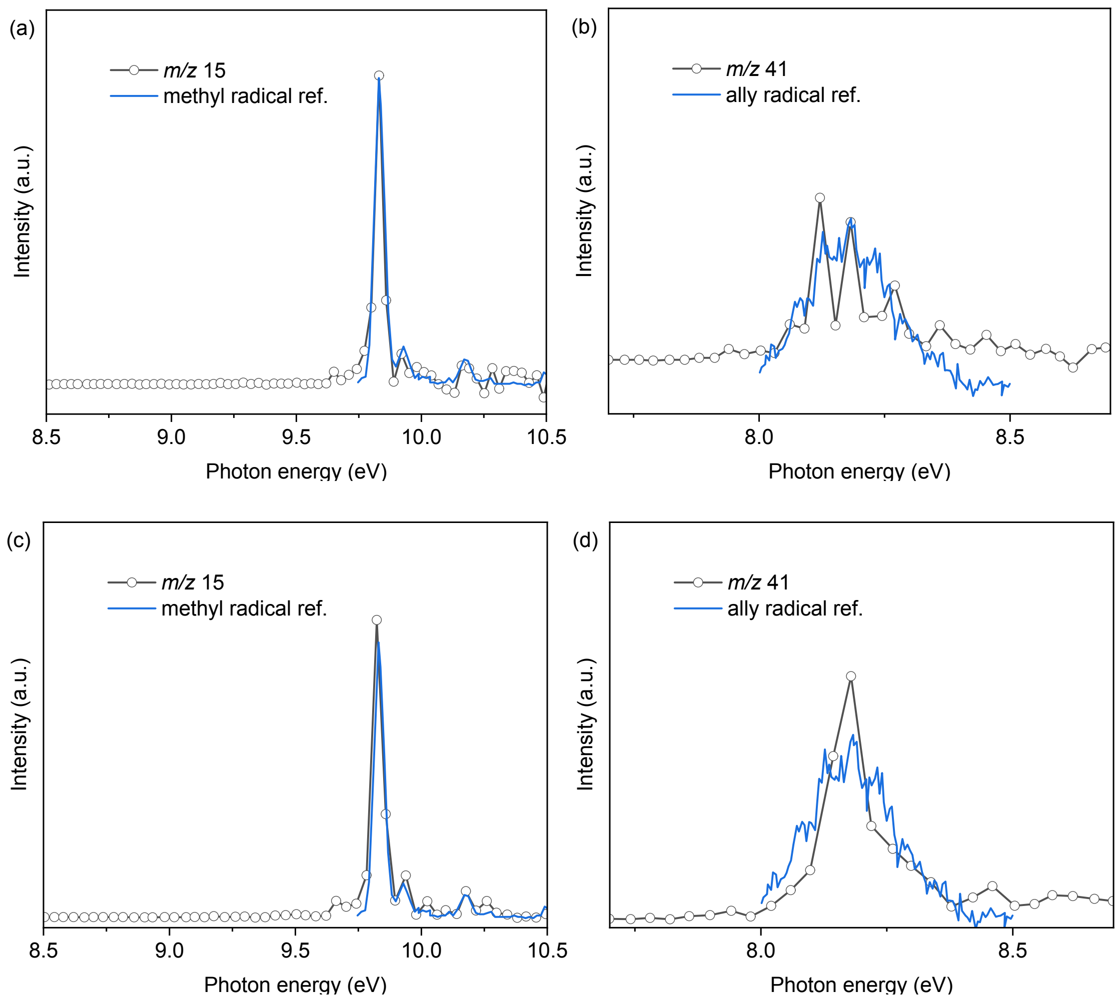

Fig. S7 ms-TPES of *m*/*z* 15 and *m*/*z* 41 in ODHP over BN at (a,b) 650 and (c,d) 700 °C.

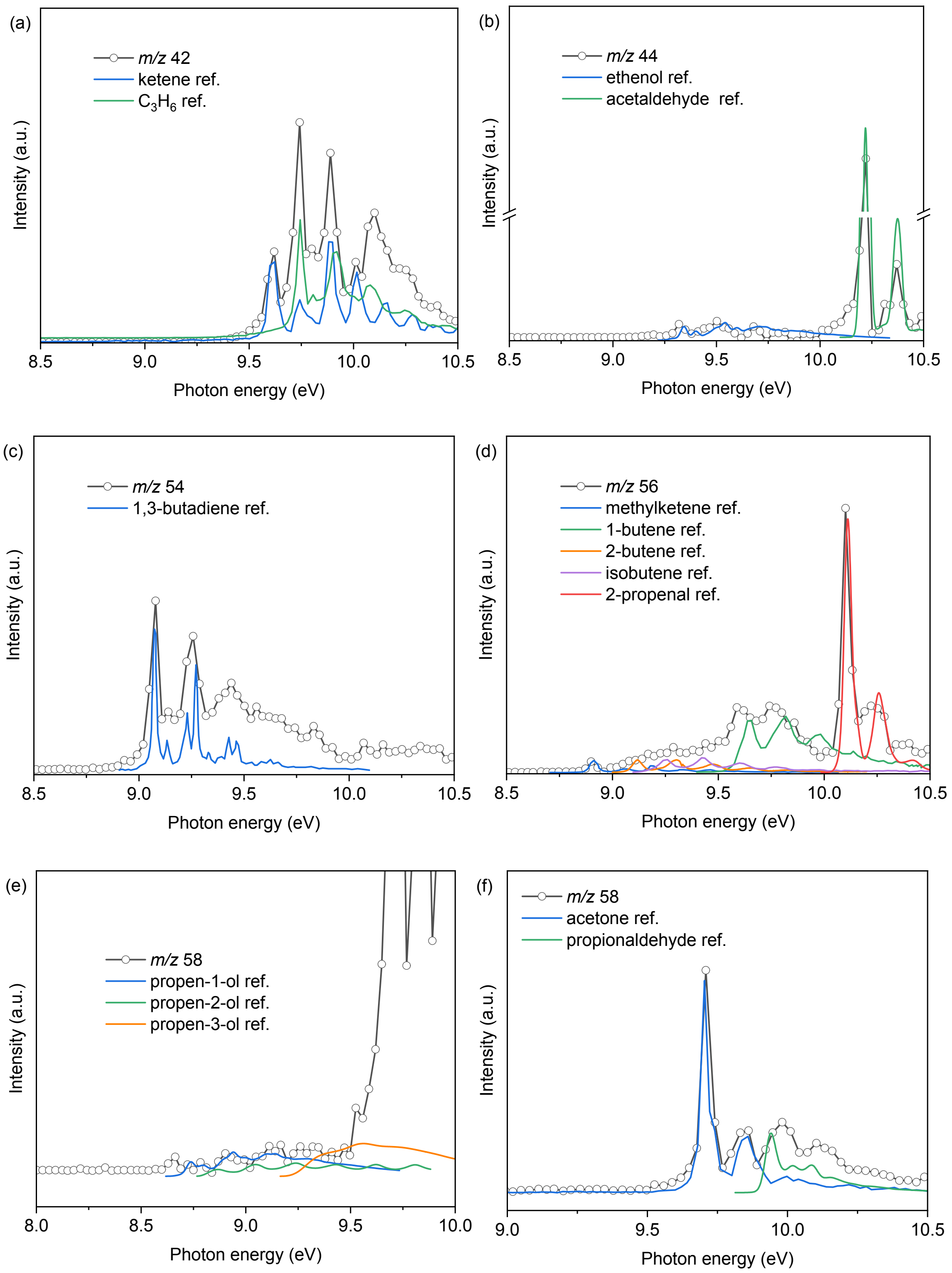

Fig. S8 ms-TPES of (a) *m*/*z* 42, (b) *m*/*z* 44, (c) *m*/*z* 54, (d) *m*/*z* 56, and (e,f) *m*/*z* 58 in ODHP over BN at 650 °C.

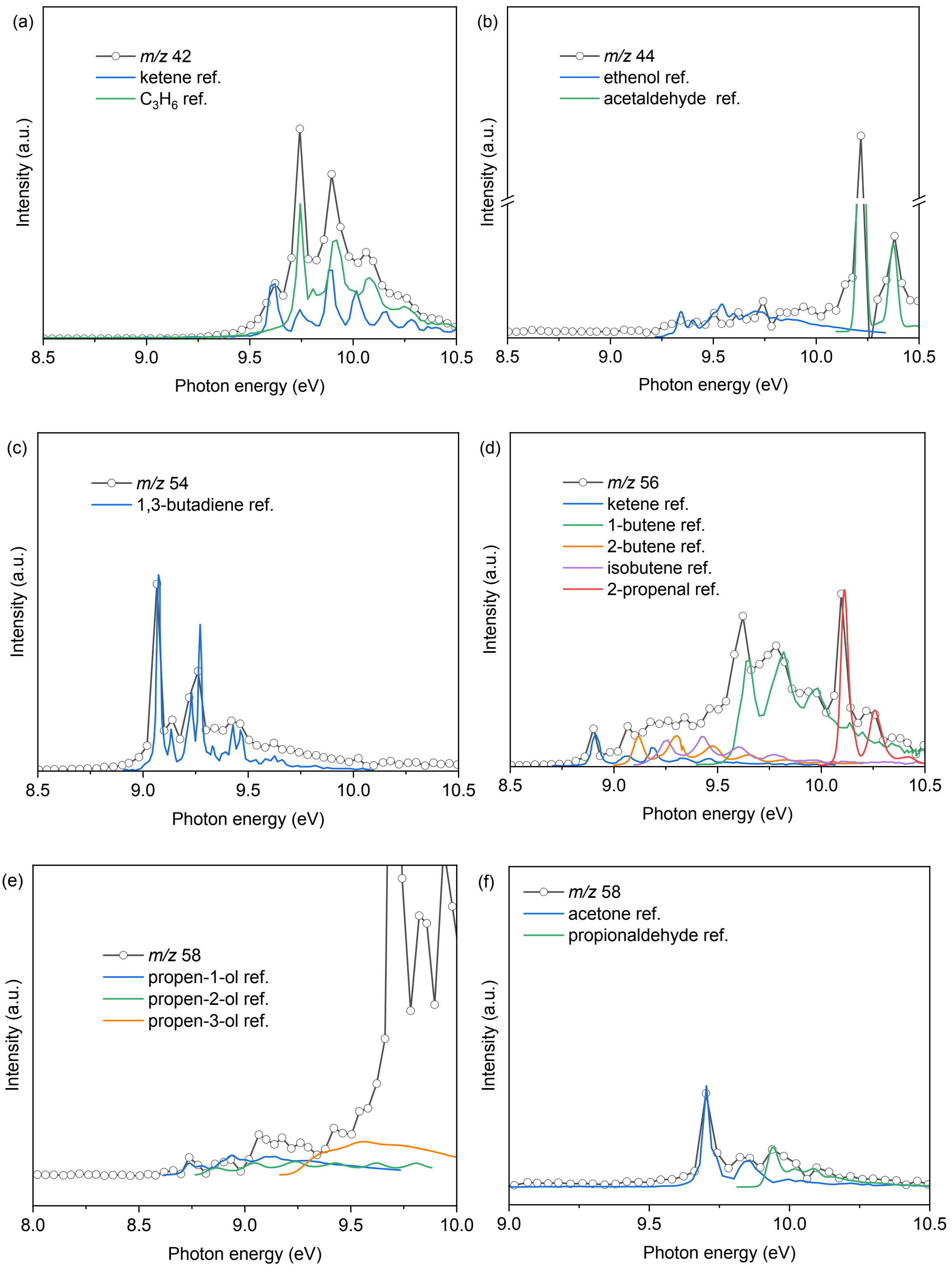

Fig. S9 ms-TPES of (a) *m*/*z* 42, (b) *m*/*z* 44, (c) *m*/*z* 54, (d) *m*/*z* 56, and (e,f) *m*/*z* 58 in ODHP over BN at 700 °C.

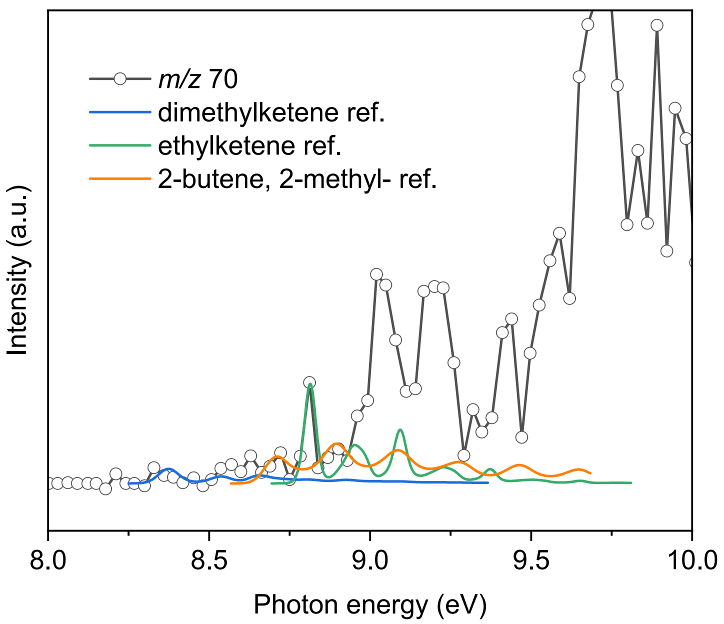

Fig. S10 ms-TPES of *m*/*z* 70 in ODHP over BN at 600 °C. The ionization energies of possible constituents listed in the NIST Chemistry Webbook are shown in Table S2. Among the C_5 isomers, only 2-methyl-2-butene has a lower ionization energy than 9 eV. Based on the FC simulation for 2-methyl-2-butene and two possible C_4 oxygenates (ethylketene and dimethylketene), the ms-TPES peak at ~8.8 eV can only be assigned to ethylketene. The low signal below 8.5 eV can be tentatively ascribed to dimethylketene. The peaks above 9 eV are attributed to different pentene isomers.

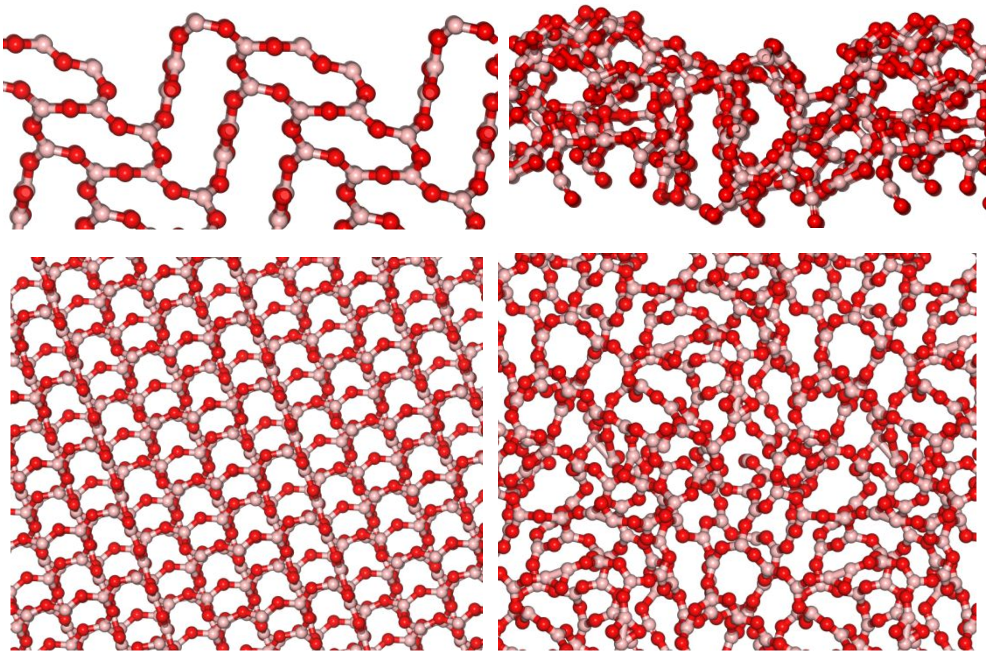

Fig. S11 The (left) crystalline B_2O_3 (101) surface and (right) disordered B_2O_3 (101) surface after ab initio molecular dynamics (AIMD) simulations at 1500–2000 K.

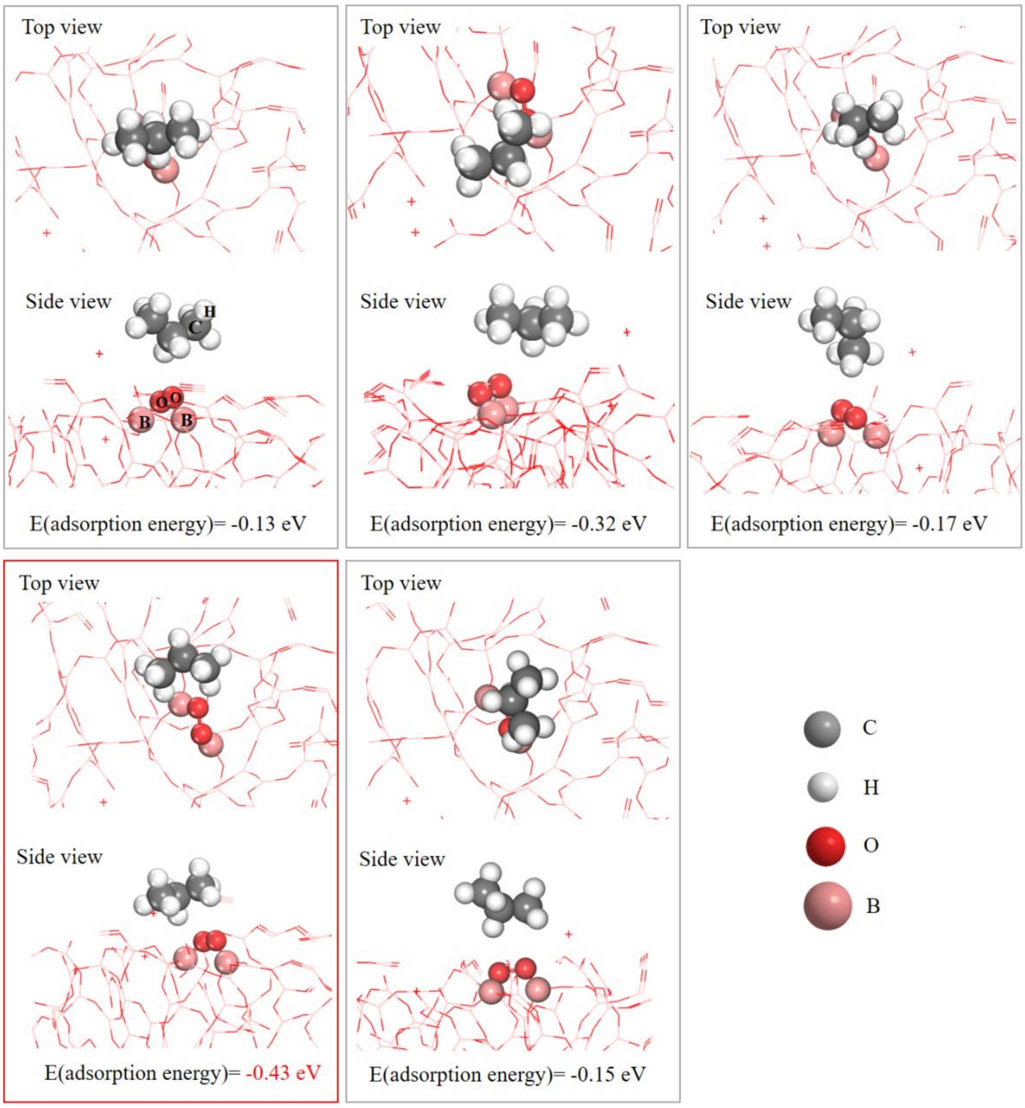

Fig. S12 Adsorption configurations of C_3H_8 on the >BO−OB< site and their corresponding adsorption energies.

C_3H_8 + >BO−OB< ⟶ >BOC_2H_5 + >$BOCH_3$ C_3H_8 + >BO−OB< ⟶ >$BOOC_2H_5$ + *CH_3

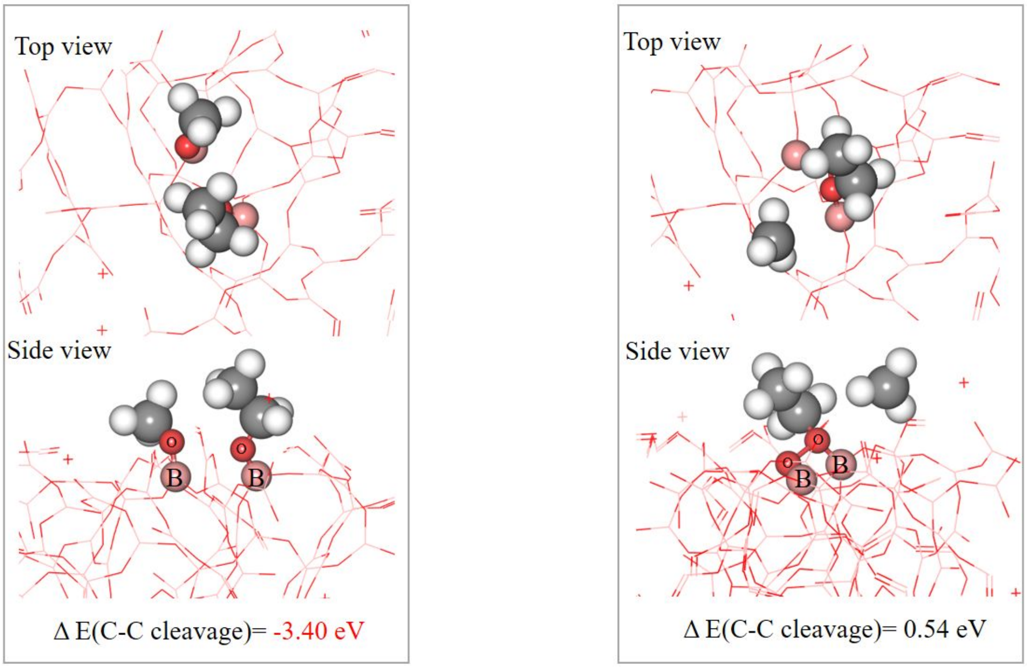

Fig. S13 Two C–C cleavage pathways of C_3H_8 on the >BO−OB< site and the corresponding reaction energies. The C–C cleavage pathway on the left is thermodynamically more favorable.

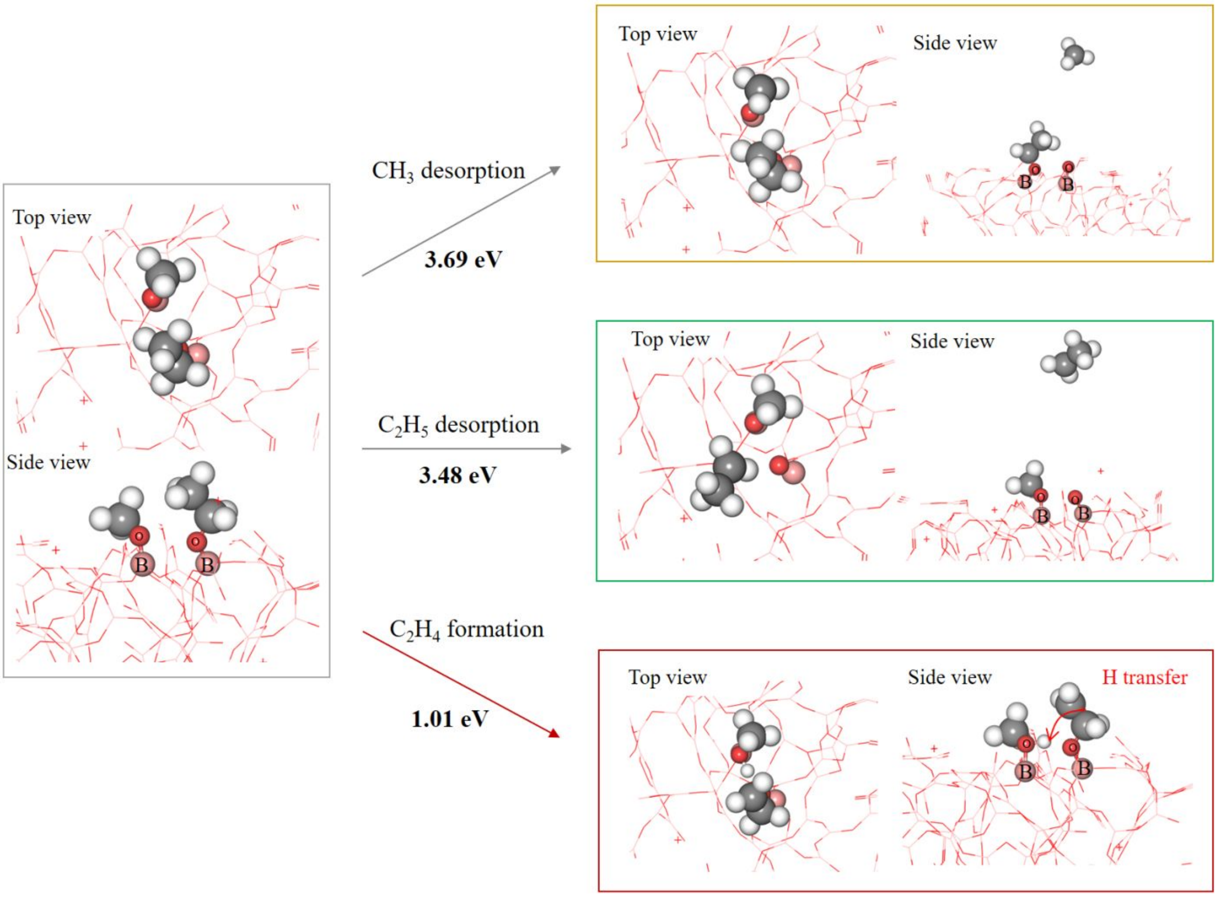

Fig. S14 Possible pathways of cracked CH_3 and C_2H_5 on the >BO−OB< site. The direct desorption of CH_3 and C_2H_5 is less favorable compared to C_2H_4 formation by H transfer.

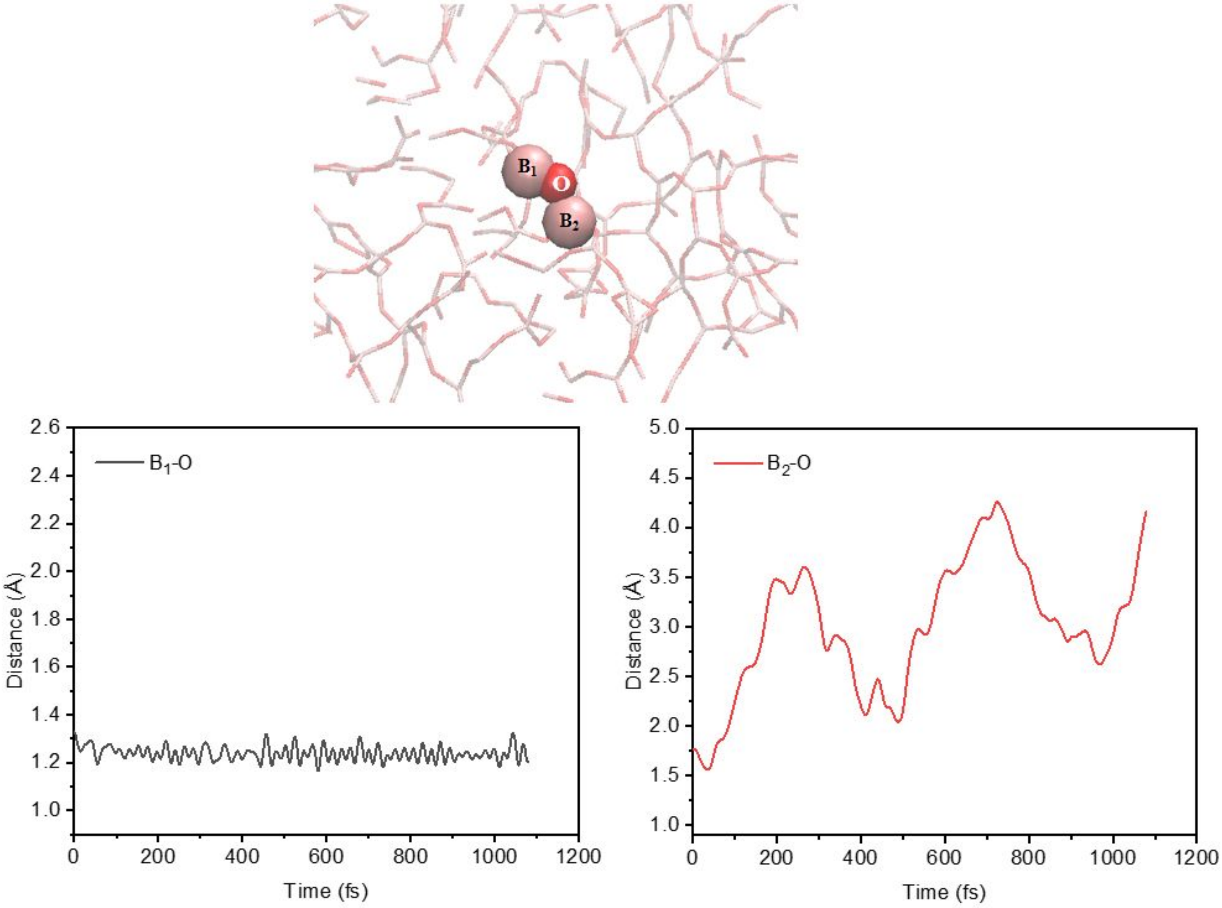

Fig. S15 Ab initio molecular dynamics (AIMD) simulations of surface BO_x at 1000 K for > 1 ps. Evolution of ⟨B_1−O⟩ and ⟨B_2−O⟩ distances shows that a >BO dangling site will be formed readily at 1000 K.

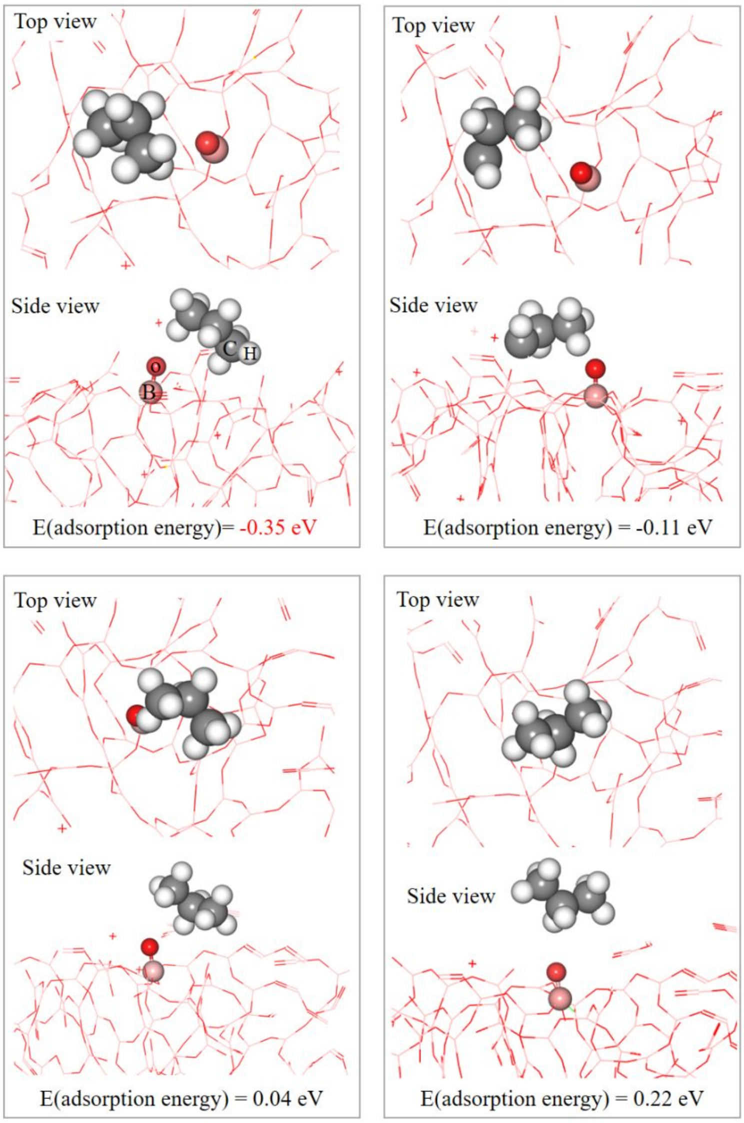

Fig. S16 The adsorption configurations of C_3H_8 on the >BO dangling site and their corresponding adsorption energies.

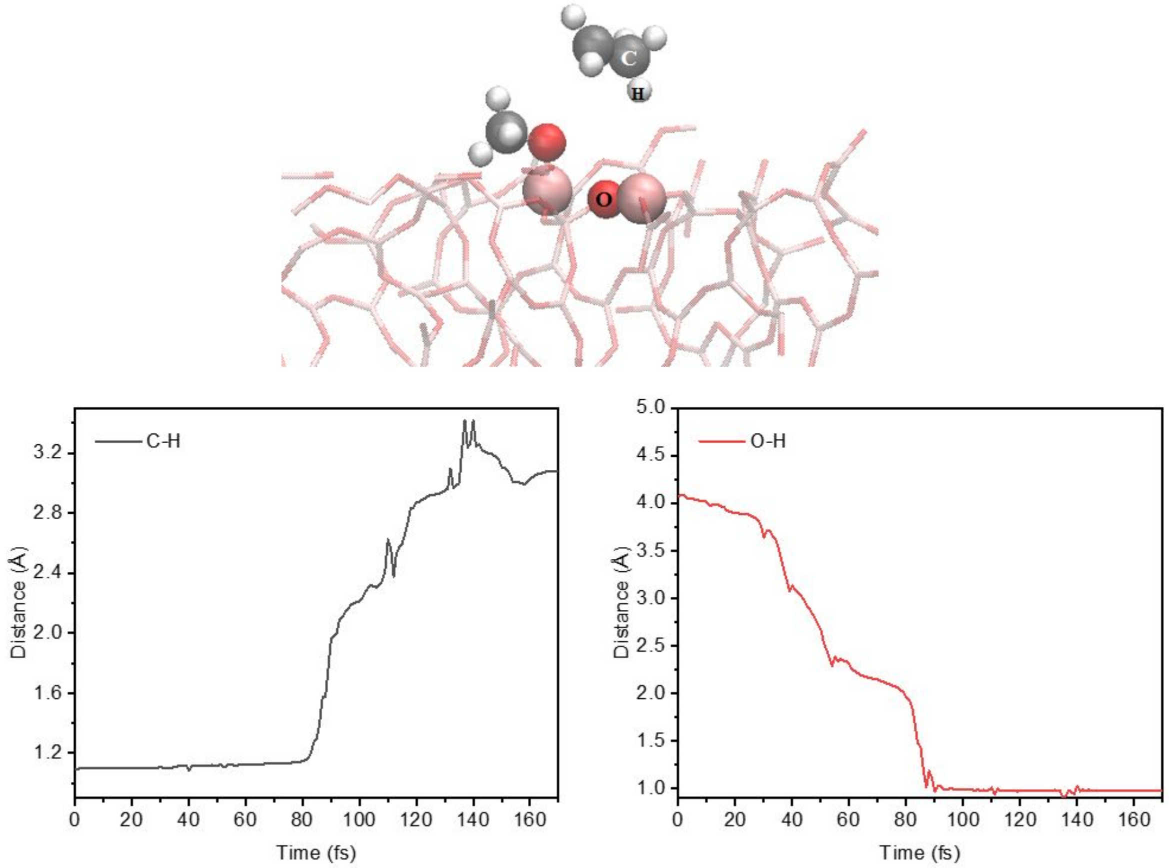

Fig. S17 Evolution of ⟨C−H⟩, ⟨O−H⟩ distances during proton transfer for the transformation of $C_2H_5^*$ to C_2H_4 species on the >BO dangling site. After C–C cleavage of C_3H_6 into >BO–CH_3^*/$C_2H_5^*$, $C_2H_5^*$ to $C_2H_4^*$ occurs spontaneously in less than 0.1 ps (100 fs) before desorption. This observation suggests that $C_2H_5^*$ is unlikely to desorb.

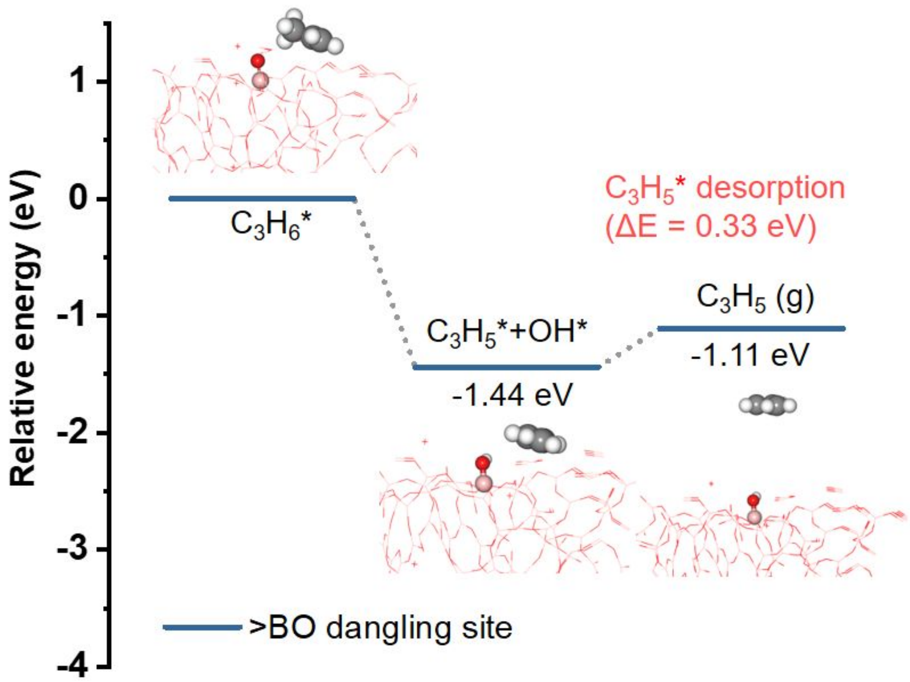

Fig. S18 DFT calculated energy profile for $C_3H_6^*$ activation to $C_3H_5^*$ process over the >BO dangling site on disordered $B_2O_3(101)$ surface. The models show the corresponding side-view of the geometries.

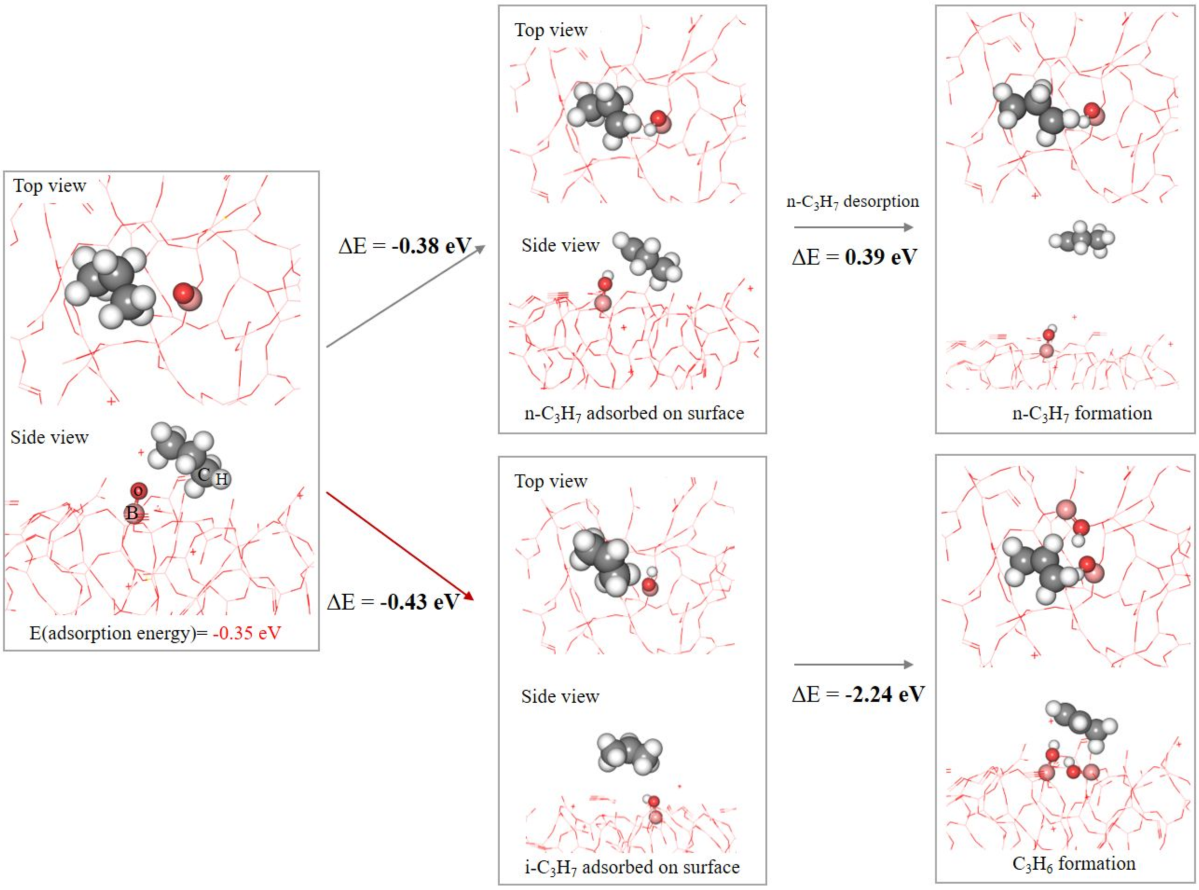

Fig. S19 Two different C–H cleavage pathways of C_3H_8 on the >BO dangling site and the corresponding reaction energies. The result shows that the *n*-C_3H_7* can desorb from the catalyst surface with a mild desorption energy of 0.39 eV. However, *i*-C_3H_7* will spontaneously transform into C_3H_6 during the structural optimization (see Fig. S20 for details).

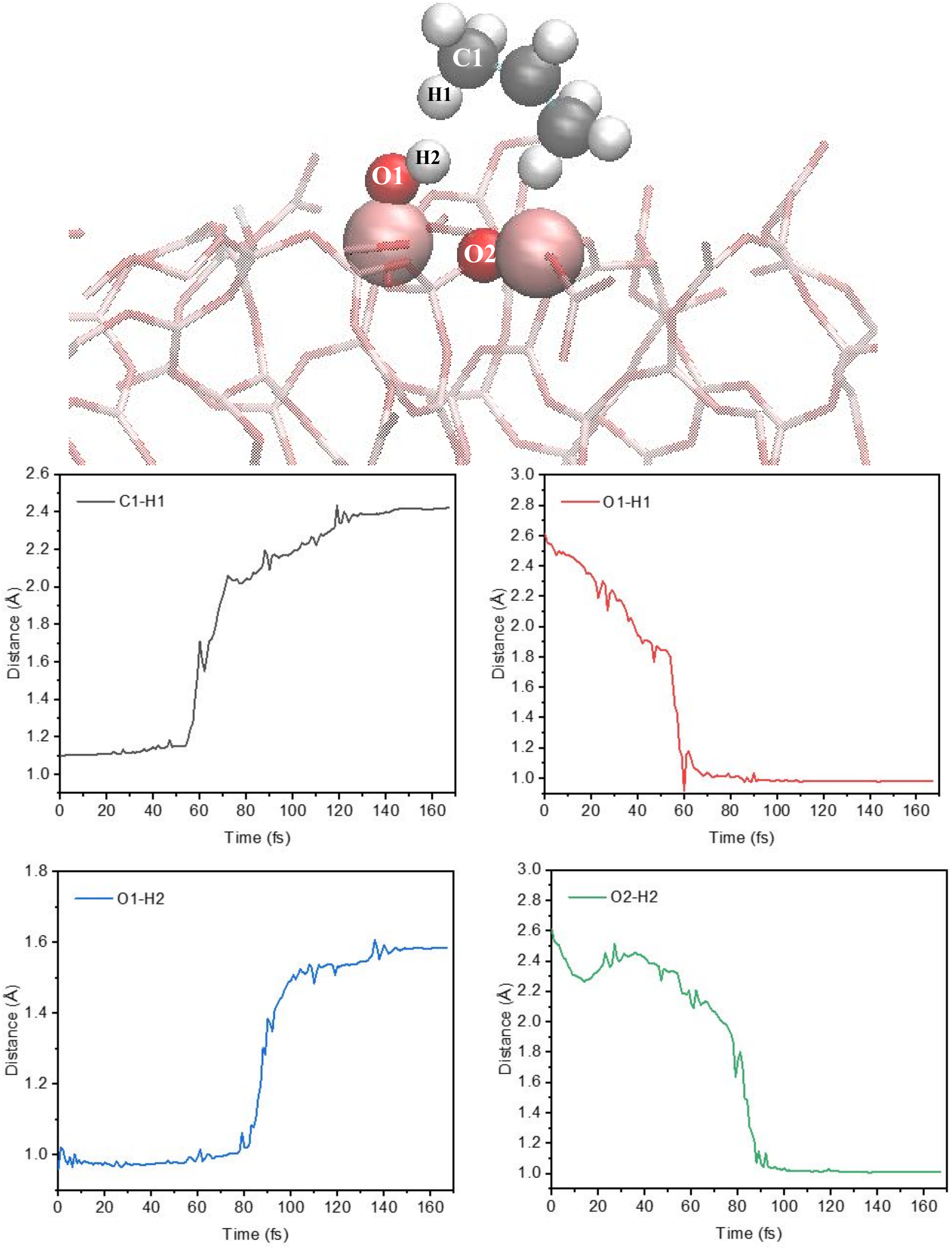

Fig. S20 Evolution of ⟨C_1−H_1⟩, ⟨O_1−H_1⟩, ⟨O_1−H_2⟩, ⟨O_2−H_2⟩ distances during H transfer during the transformation of *i*-C_3H_7* to C_3H_6. The transformation includes two sequential H transfer steps. First, *i*-C_3H_7* adsorbs on the O1H2* via an O1H1 interaction. Second, the H2 atom linked to O1 of the >BO dangling site transfers to the nearby O2. Eventually, C1–H1 cleavage occurs, generating C_3H_6. The overall transformation from *i*-C_3H_7* to C_3H_6 occurs spontaneously in less than 0.1 ps (100 fs). These observations suggest that *i*-C_3H_7* is very easy to transform to C_3H_6 over the >BO dangling site.

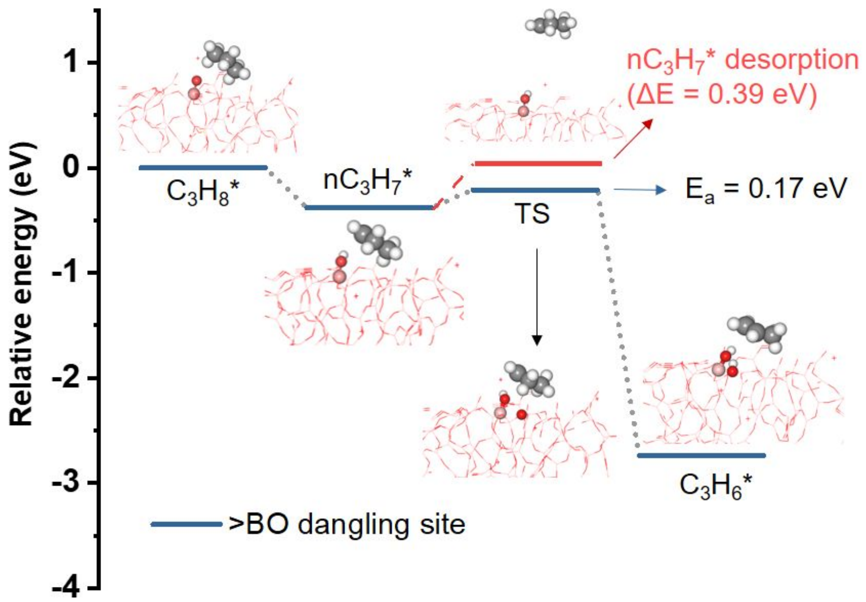

Fig. S21 DFT calculated energy profile for C–H cleavage over the >BO dangling site on disordered $B_2O_3(101)$ surface. The models show the corresponding side-view of the geometries.

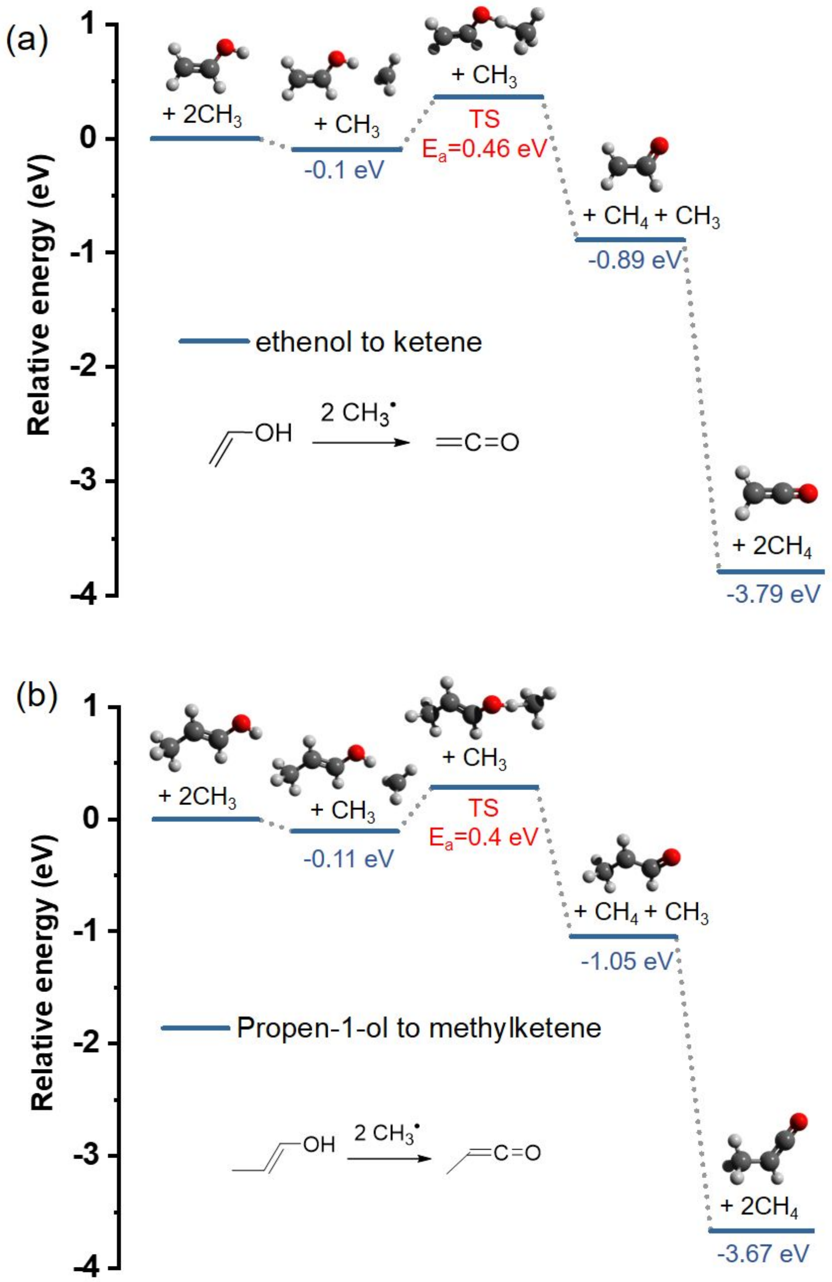

Fig. S22 G4 calculated energy profile for (a) ethenol to ketene and (b) propen-1-ol to methylketene routes with two $CH_3^{\bullet}$ radicals in the gas-phase. The models show the corresponding optimized geometries along the reaction coordinate.

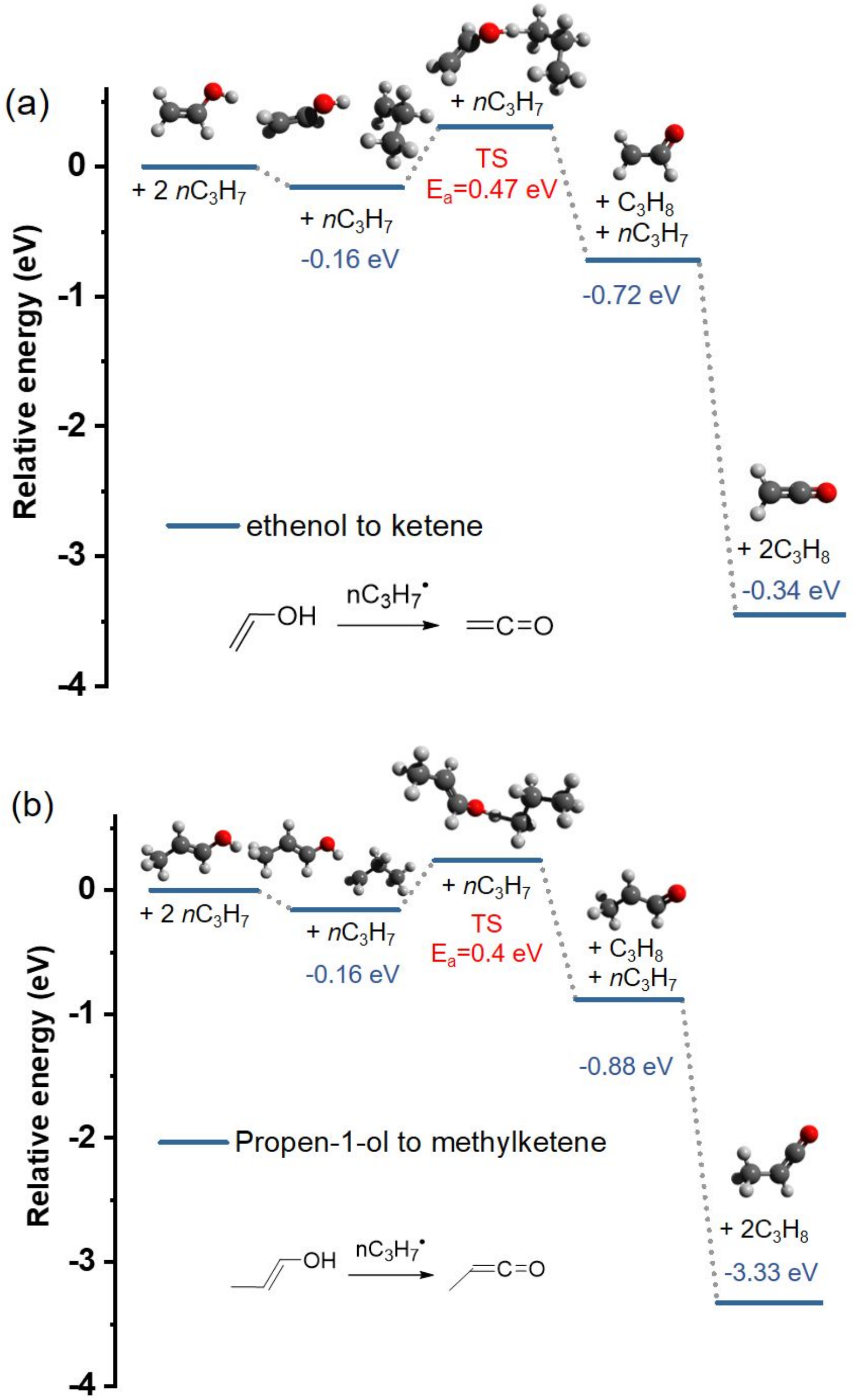

Fig. S23 G4 calculated energy profile for (a) ethenol to ketene and (b) propen-1-ol to methylketene routes with two n-$C_3H_7^{\bullet}$ radicals in the gas-phase. The models show the corresponding optimized geometries along the reaction coordinate.

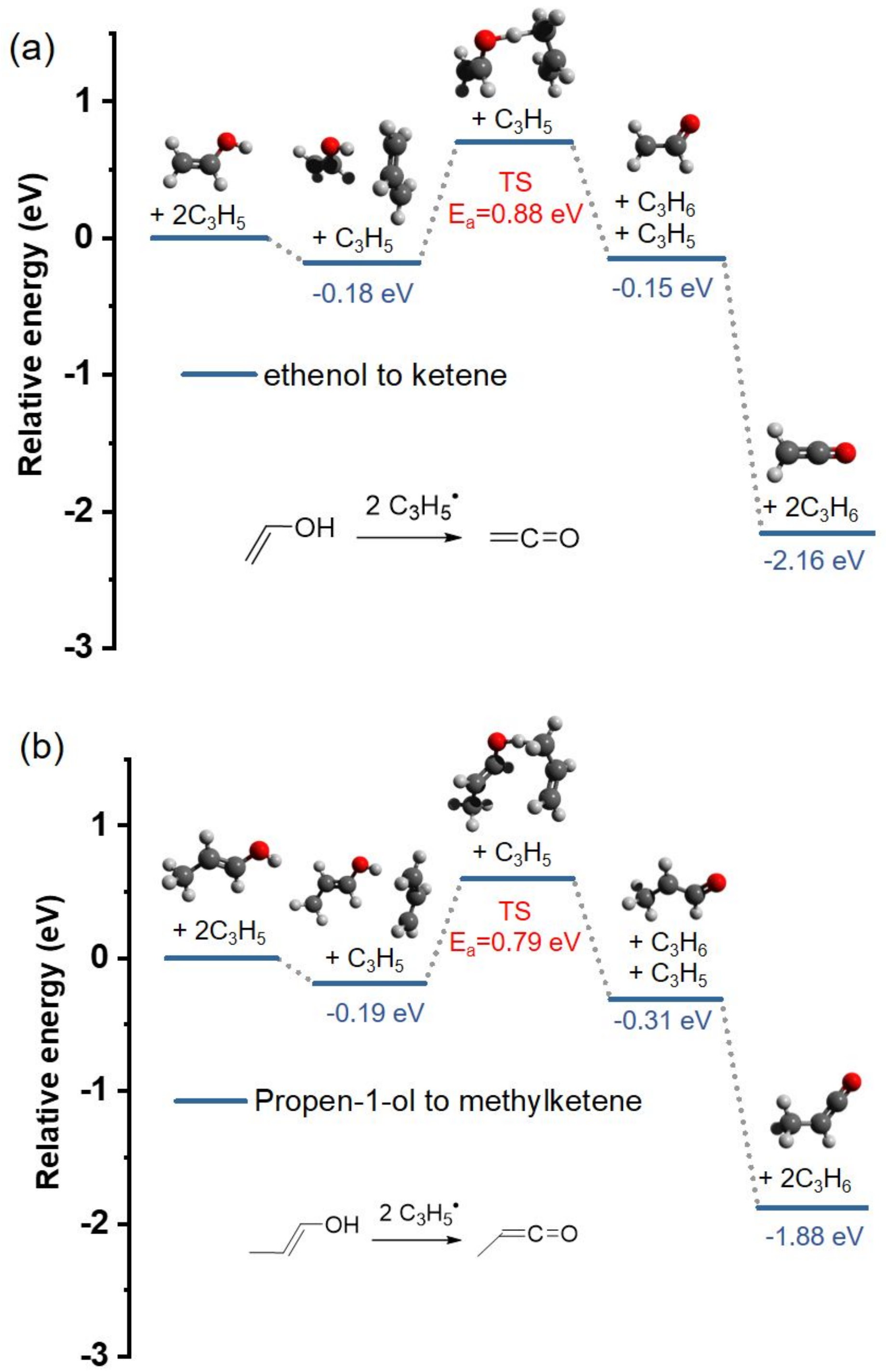

Fig. S24 G4 calculated energy profile for (a) ethenol to ketene and (b) propen-1-ol to methylketene routes with two $C_3H_5^•$ radicals in the gas-phase. The models show the optimized geometries along the reaction coordinate.

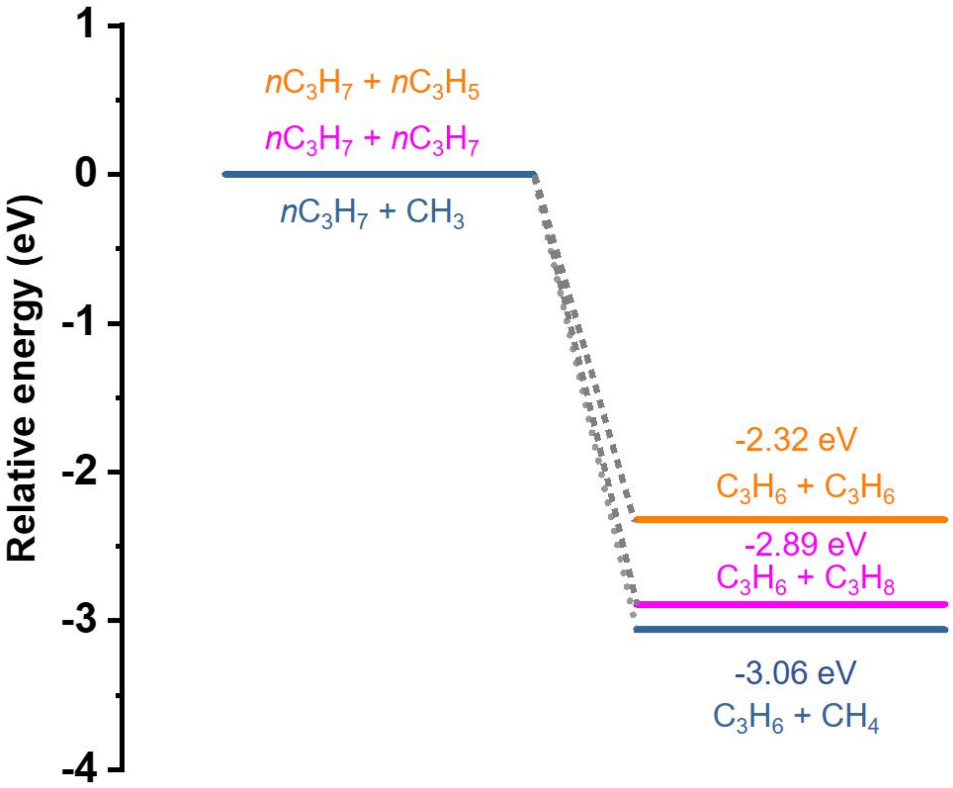

Fig. S25 G4 calculated energy profile for *n*-$C_3H_7^{\bullet}$ conversion with *n*-$C_3H_7^{\bullet}$, $C_3H_5^{\bullet}$, and CH_3 in the gas phase.

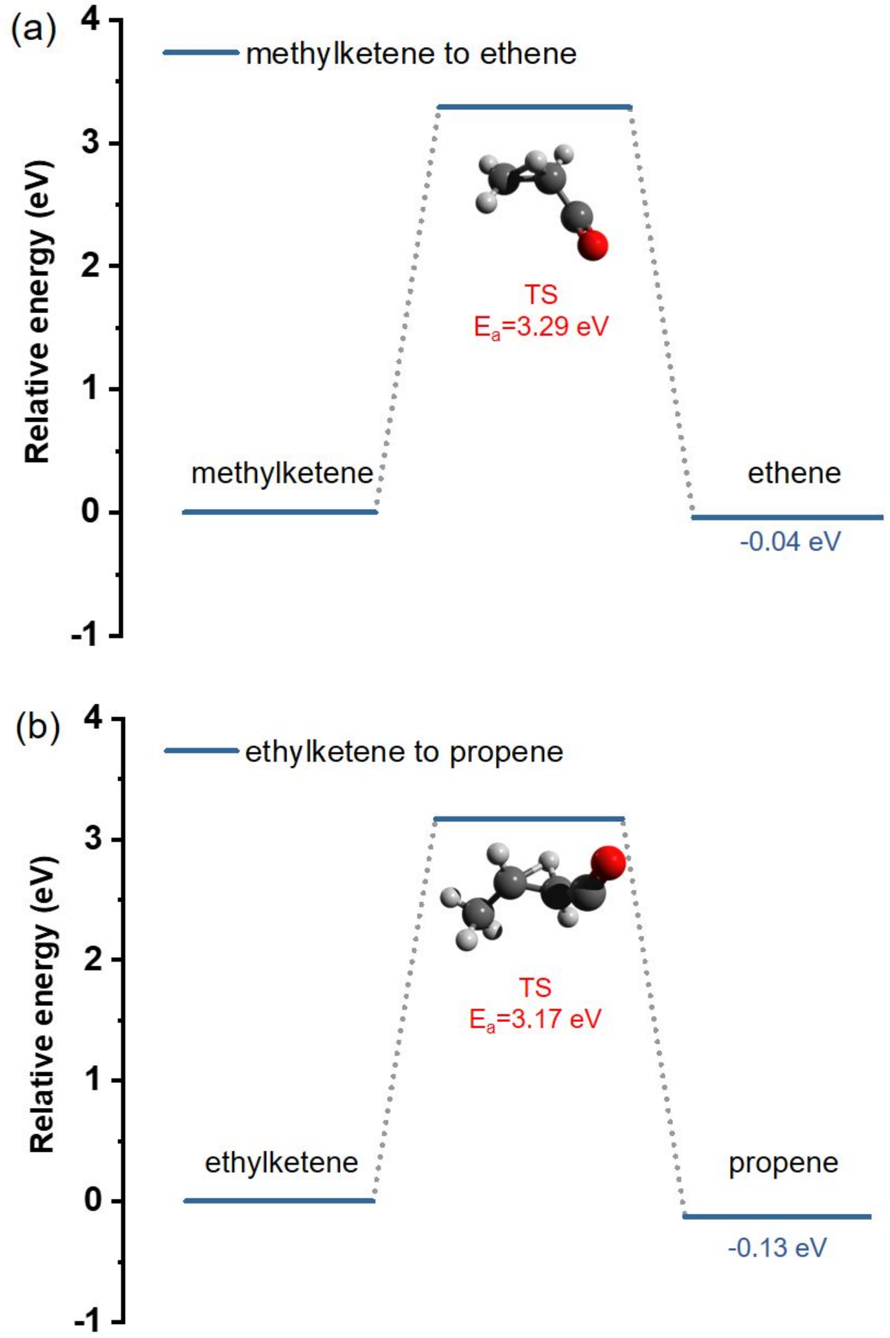

Fig. S26 G4 calculated energy profile for (a) methylketene to ethene and (b) ethylketene to propene routes. The models show the optimized geometries along the reaction coordinate. The CO co-product is not shown. The high activation energies suggest that unimolecular decarbonylation does not take place in the gas phase, but is, like in the MTO mechanism, a surface-assisted process.[17]

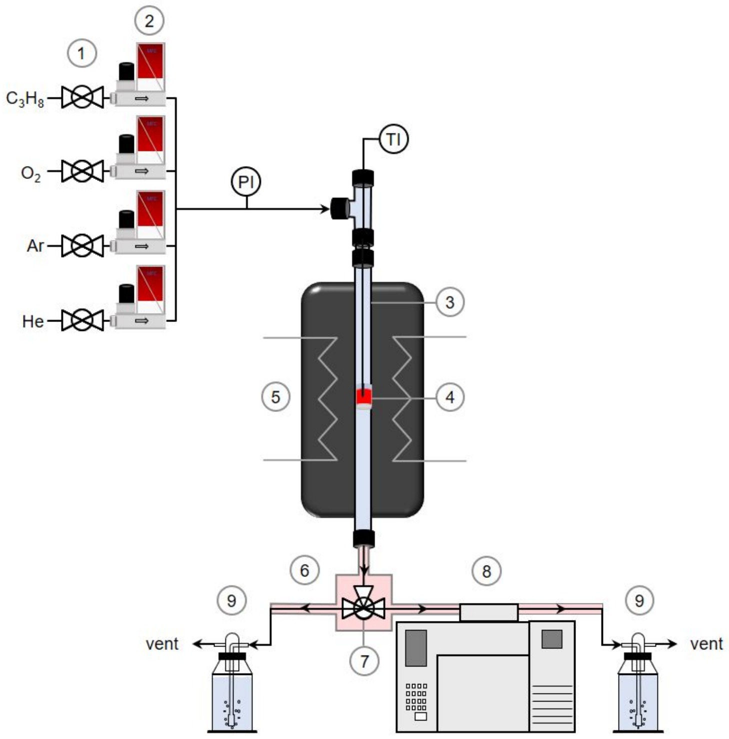

Fig. S27 Flowsheet of the laboratory set-up used for catalytic evaluation of oxidative propane dehydrogenation. 1: two-way on/off valves, 2: mass flow controllers, 3: quartz reactor, 4: catalyst bed, 5: oven, 6: heat tracing (red background), 7: three-way sampling valve, 8: gas chromatograph coupled to a mass spectrometer (GC-MS), 9: H_2O scrubbers, PI: pressure indicator, and TI: temperature indicator.

References

[1] J. Tian, J. Li, S. Qian, Z. Zhang, S. Wan, S. Wang, J. Lin, Y. Wang, *Appl. Catal. A: Gen.* **2021**, *623*, 118271.

[2] A. Cesarini, S. Mitchell, G. Zichittella, M. Agrachev, S. P. Schmid, G. Jeschke, Z. Pan, A. Bodi, P. Hemberger, J. Pérez-Ramírez, *Nat. Catal.* **2022**, *5*, 605-614

[3] A. Bodi, P. Hemberger, T. Gerber, B. Sztáray, *Rev. Sci. Instrum.* **2012**, *83*, 083105.

[4] B. Sztáray, K. Voronova, K. G. Torma, K. J. Covert, A. Bodi, P. Hemberger, T. Gerber, D. L. Osborn, *J. Chem. Phys.* **2017**, *147*, 013944.

[5] F. Holzmeier, M. Lang, I. Fischer, X. Tang, B. Cunha de Miranda, C. Romanzin, C. Alcaraz, P. Hemberger, *J. Chem. Phys.* **2015**, *142*, 184306.

[6] J. VandeVondele, M. Krack, F. Mohamed, M. Parrinello, T. Chassaing, J. Hutter, *Comput. Phys. Commun.* **2005**, *167*, 103-128.

[7] J. P. Perdew, K. Burke, M. Ernzerhof, *Phys. Rev. Lett.* **1996**, *77*, 3865.

[8] S. Goedecker, M. Teter, J. Hutter, *Phys. Rev. B* **1996**, *54*, 1703.

[9] S. Grimme, J. Antony, S. Ehrlich, H. Krieg, *J. Chem. Phys.* **2010**, *132*, 154104.

[10] J. M. Venegas, Z. Zhang, T. O. Agbi, W. P. McDermott, A. Alexandrova, I. Hermans, *Angew. Chem. Int. Ed.* **2020**, *59*, 16527-16535.

[11] X. Zhang, R. You, Z. Wei, X. Jiang, J. Yang, Y. Pan, P. Wu, Q. Jia, Z. Bao, L. Bai, *Angew. Chem. Int. Ed.* **2020**, *59*, 8042-8046.

[12] N. W. Assaf, M. De La Pierre, M. K. Altarawneh, M. W. Radny, Z.-T. Jiang, B. Z. Dlugogorski, *The J. Phys. Chem. C* **2017**, *121*, 11346-11354.

[13] G. Henkelman, B. P. Uberuaga, H. Jónsson, *J. Chem. Phys.* **2000**, *113*, 9901-9904.

[14] S. Nosé, *J. Chem. Phys.* **1984**, *81*, 511-519.

[15] M. Frisch, G. Trucks, H. Schlegel, G. Scuseria, M. Robb, J. Cheeseman, G. Scalmani, V. Barone, G. Petersson, H. Nakatsuji, Gaussian 09 (Gaussian, Inc., Wallingford, CT, **2009**).

[16] L. A. Curtiss, P. C. Redfern, K. Raghavachari, *J. Chem. Phys.* **2007**, *126*, 084108.

[17] X. Wu, Z. Zhang, Z. Pan, X. Zhou, A. Bodi, P. Hemberger. *Angew. Chem. Int. Ed.* **2022**, *61*, e202207777.

[18] I. Derbali, H.R. Hrodmarsson, M. Schwell, Y. Bénilan, L. Poisson, M. Hochlaf, M.E. Alikhani, J.C. Guillemin, E.L. Zins, *Phys. Chem. Chem. Phys.* **2020**, *22*, 20394-20408.

[19] D. Krüger, P. Oßwald, M. Köhler, P. Hemberger, T. Bierkandt, Y. Karakaya, T. Kasper, *Combust. Flame* **2018**, *191*, 343-352.

[20] D.M.P. Holland, M.A. MacDonald, M.A. Hayes, P. Baltzer, B. Wannberg, M. Lundqvist, L. Karlsson, W. Von Niessen, *J. Phys. B-AT Mol. Opt.* **1996**, *29*, 3091.

[21] P. Hemberger, X. Wu, Z. Pan, A. Bodi. *J. Phys. Chem. A* **2022**, *126*, 2196-2210.

[22] J. Dyke, N. Jonathan, E. Lee, A. Morris. *J. Chem. Soc., Faraday Trans 2: Mol. Chem. Phys.* **1976**, *72*, 1385-1396.

[23] https://webbook.nist.gov/chemistry/form-ser/
